# Insights into planktonic food-web dynamics through the lens of size and season

**DOI:** 10.1038/s41598-024-52256-4

**Published:** 2024-01-19

**Authors:** Carolina Giraldo, Pierre Cresson, Kirsteen MacKenzie, Virginie Fontaine, Christophe Loots, Alice Delegrange, Sébastien Lefebvre

**Affiliations:** 1 IFREMER, HMMN – Unité halieutique Manche‐Mer du Nord, 62200 Boulogne sur mer, France; 2 Univ. Lille, CNRS, Univ. Littoral Côte d’Opale, IRD, UMR 8187 – LOG – Laboratoire d’Océanologie et de Géosciences, 59000 Lille, France

**Keywords:** Ecology, Marine biology, Ecological networks

## Abstract

Knowledge of the trophic structure and variability of planktonic communities is a key factor in understanding food-web dynamics and energy transfer from zooplankton to higher trophic levels. In this study, we investigated how stable isotopes of mesozooplankton species varied seasonally (winter, spring, autumn) in relation to environmental factors and plankton size classes in a temperate coastal ecosystem. Our results showed that spring is characterized by the strongest vertical and size-structured plankton food-web, mainly fueled by the phytoplankton bloom. As a result, spring displayed the largest isotopic niche space and trophic divergence among species. On the contrary, both pelagic and benthic-derived carbon influenced low productive seasons (winter and autumn), resulting in more generalist strategies (trophic redundancy). Stable isotope mixing models were used to explore how different seasonal structures influenced the overall food web up to predatory plankton (i.e., mysids, chaetognaths, and fish larvae). Different feeding strategies were found in spring, with predators having either a clear preference for larger prey items (> 1 mm, for herring and dab larvae) or a more generalist diet (sprat and dragonets larvae). During low productive seasons, predators seemed to be more opportunistic, feeding on a wide range of size classes but focusing on smaller prey. Overall, the food-web architecture of plankton displayed different seasonal patterns linked to components at the base of the food web that shaped the main energy fluxes, either from phytoplankton or recycled material. Additionally, these patterns extended to carnivorous plankton, such as fish larvae, emphasizing the importance of bottom-up processes.

## Introduction

Lower trophic levels organisms, and in particular zooplankton, are key components of marine food-webs and play an essential role in nutrient cycles, transfer of energy to upper trophic levels, and fish recruitment trough larval fish survival^1–3^. Ecosystem services provided by zooplankton are not only a function of overall abundances but are also influenced by species composition and functional diversity^4^. Most plankton species, and in particular copepods, are considered omnivorous, relying directly or indirectly on primary producers as food^5^. Seasonal variation in the abundance or quality of phytoplankton therefore affects consumers’ feeding behavior^6^ both in terms of opportunistic feeding (on the most abundant prey) and also selectivity or specialization (based on biological stoichiometry) for prey that matches consumers’ metabolic needs^7^. Seasonal fluctuations are thus expected to be one of the main drivers of variation within zooplankton food-webs^8^ that might transfer up to predatory plankton and zooplanktivorous fish. However, capturing full seasonal variations over relatively large spatial gradients would require a consistent sampling throughout the year, which is rarely the case. Most currently available data and ecosystemic models on food-webs thus consider zooplankton a single group, regardless of species, ecological role, or size (but see e.g. ^9,10^). Higher resolution data on the seasonal dynamics and trophic interactions among planktonic species (including fish larvae) therefore remain necessary to improve the accuracy of ecosystem-based models^11–13^ and our understanding of ecosystem functioning and response to environmental drivers.

The majority of ecological processes are linked to species' size, which is considered a 'master trait' in ecology, influencing species abundance, rates of production, metabolism, and mortality rates^14,15^. This trait also informs potential predator–prey interactions^16^, and size-based approaches have been widely used to infer marine food-web structures, including plankton studies^9,17^. In combination with stable isotopes, used to trace the origin (carbon δ^13^C) and transfer (nitrogen δ^15^N) of organic matter^18^, analysis by size-classes has primarily been used to shed light on the vertical structure (i.e. number of trophic levels) of food-webs^19,20^. Few studies have reported data on major planktonic taxonomic groups, mainly due to the difficulties on identifying and sorting sufficient individuals for laboratory analysis. However, available studies highlight important among-species variation (i.e. trophic divergence, representing varying or divergent trophic roles among different species). These studies indicate that the zooplankton is characterized by a wide spectrum of feeding strategies, resulting on at least three trophic levels, from filter feeders to carnivorous plankton including fish larvae^21,22^. This is confirmed by the increase of δ^15^N in size-classes of plankton, which is positively linked to an organism size, illustrating size-related consumption modes in marine plankton food-webs^23^. Seasonal and spatial variations in the quantity and quality of primary production are therefore expected to impact the size-structure of the zooplankton food-web^10,23–25^. The strength (i.e. slope) of the relationship between δ^15^N values and plankton’ size-classes is typically higher during productive periods (i.e. spring) of mesotrophic temperate coastal waters^23^. Romero-Romero et al. (2019) hypothesized that this change in slope was dependent on the main seasonal trophic pathways in the zooplankton food-web (phytoplankton vs. detritus or new vs. regenerated production). In contrast, in oligotrophic waters, higher trophic levels (i.e. higher slope of the δ^15^N values and size-classes of plankton relationship) were found in less productive locations due to the higher number of trophic mediators in the microbial food-web^24,25^. Overall, seasonal variability in planktonic food webs appears to be closely linked to fluctuations in flows through microbial food web complexes. These connections are often overlooked due to the challenges associated with quantifying trophic steps in complex natural assemblages of interacting microbes^26^.

Spatio-temporal variations on the primary productivity of coastal ecosystems, as well as zooplankton species identity and their degree of omnivory^27^, are key drivers of size-structure at the base of the food-web. The impact of different zooplankton size-structures (different food-web architectures) on foraging patterns of predatory plankton such as fish larvae remains an open question. Fish larvae have sufficiently long isotopic turnover times to reflect seasonal changes in their diet^17^. Optimal foraging, a strategic feeding behavior where species aim to maximize their energy intake while minimizing the energy spent during foraging by consuming the largest biomass per unit effort, seems to be the dominant pattern in zooplanktivorous fish^28^. However, some species are known for their strong selectivity patterns in terms of both species composition or size classes^29–32^, which might result in lower competition from resource partitioning among predators. For species with a strong selectivity for specific size-classes such as *Clupea harengus* herring larvae, deviating from the optimal relative prey size (prey size divided by predator size) regardless of the available prey biomass has consequences on fish larvae growth^32^. The causal link between optimal foraging, prey size and abundance is, therefore, more difficult to decipher in fish larvae and might change depending on the specific phenology (life cycle timing, seasonal patterns) and feeding strategies unique to each species.

In this study, we investigated the seasonal dynamics of plankton food-web size-structure in a coastal ecosystem, the Eastern English Channel and the Southern Bight of the North Sea Sea (hereafter referred to as EEC for sake of simplicity), by combining data on species composition, size, and stable isotopic composition for 18 taxa of mesozooplankton and 13 taxa of fish larvae collected in winter, spring, and autumn. More specifically, we aimed to: (1) explore how the planktonic food-web structure and functioning (size-structure) respond to changes in productivity and environmental drivers, and (2) determine how seasonal variations influence food-web topology and the main energy fluxes for predatory plankton, particularly fish larvae.

## Materials and methods

### Study area

**Figure 1:**
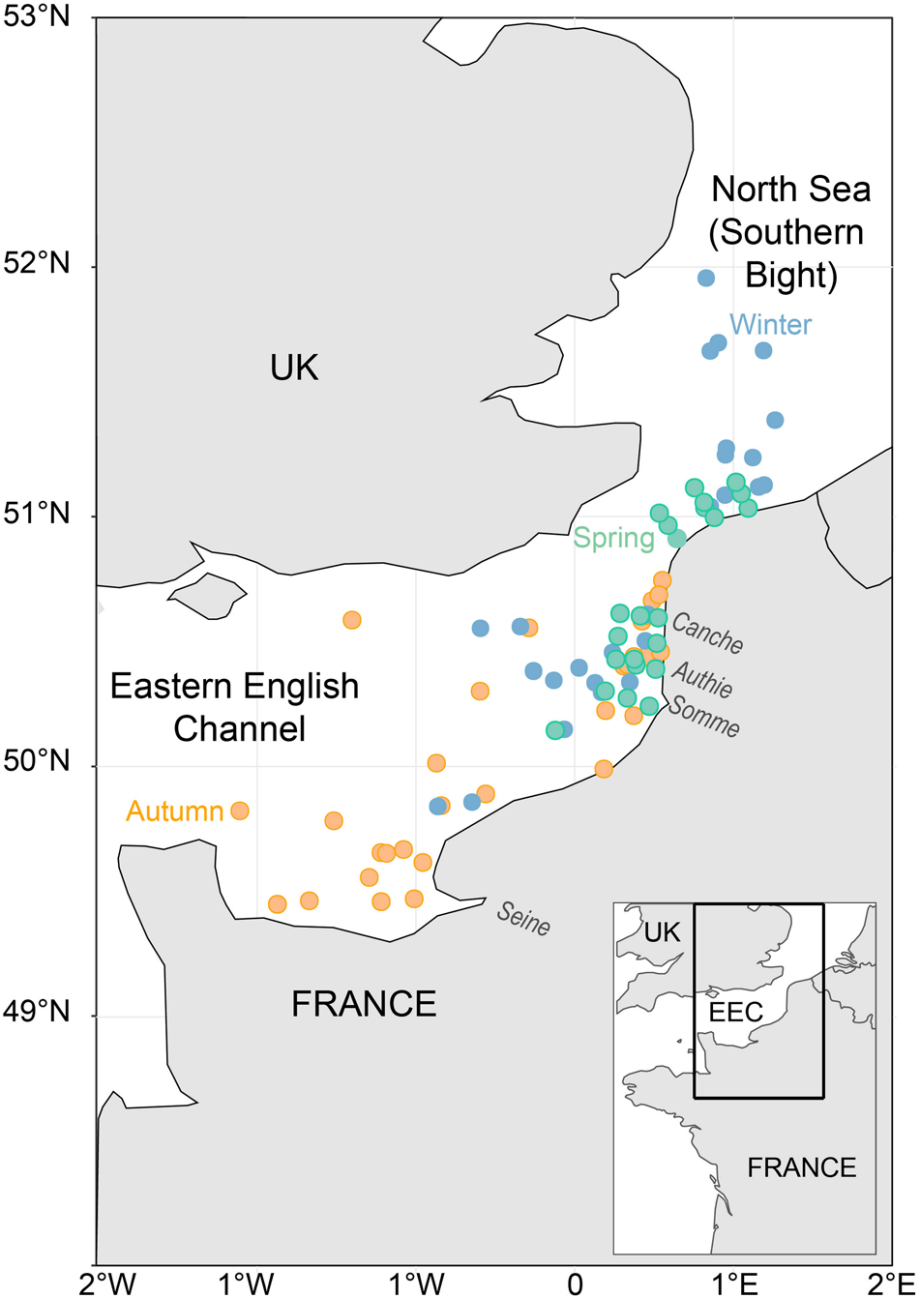
Study area including the Eastern English Channel and Southern Bight of the North Sea. Sampling stations in winter (blue), spring (green) and autumn (yellow) and main rivers along the French coast are indicated.

The Eastern English Channel and Southern Bight of the North are epicontinental seas bordered by France, Belgium, and England (Fig. 1) subject to high anthropogenic pressures (marine traffic, fishing activities, gravel extractions^33,34^). This area is very shallow, and highly productive, with strong vertical and horizontal mixing, and a seasonal temperature gradient^35^ The area has been extensively studied for more than a century to advise management decisions relating to environmental health, sustainable use of resources, and conservation^36^. With the objective of an ecosystem-based management, a wide range of available data on both abiotic (e.g., tidal hydrodynamics, sediments, Chl a) and biotic factors (e.g., species abundance, distribution and composition) have been used to implement ecosystem-based models in the EEC^37,38^. This holistic view of the ecosystem requires knowledge of species interactions in terms of predator–prey relationships together with food-web structure and functioning. For the most part, these interactions are well documented and informed for commercial fish species, but a lack of understanding remains for lower trophic levels^12,13^. Bottom-up effects, however, have recently been identified in the EEC as one of the most important drivers of variation for outputs in ecosystem-based models, affecting ecosystem dynamics and changes in biomass across all functional groups and trophic levels^39^. Similarly, studies on the community composition in the North Sea have identified that zooplankton composition and abundance are main bottom-up drivers of ecosystem dynamics^40,41^. In the EEC, spatial data comes primarily from fisheries-oriented oceanographic surveys that usually neglect the zooplankton compartment, or from dedicated ichthyoplankton surveys (e.g., International herring larvae survey, IHLS), which are restricted in time to just one season^42^. Seasonal dynamics of zooplankton remain largely uninvestigated in the EEC, with data only available from the French coastal station of Gravelines^43–45^.

### Sampling procedures

Samples were collected opportunistically from 2017 to 2019 in winter (January to February from the International Bottom Trawl Surveys, https://doi.org/10.18142/17), spring (March to May 2017, from the REIVE I and II surveys, 10.17600/17010400, and PHYCO surveys 10.17600/17010500) and autumn (September to October 2017 to 2019, from the Channel Ground Fish Surveys https://doi.org/10.18142/11) (Fig. 1). Environmental data for each survey and additional details on sampling protocols can be accessed through the survey's DOI. Briefly, environmental parameters displayed a strong seasonal pattern with temperatures ranging from a minimum of 6.5 °C in winter to a maximum of 18 °C in autumn. Salinity was rather stable throughout the seasons (~ 34) with minimum values found at stations close to estuary mouths (the Seine, Somme, Authie, and Canche estuaries). Correspondingly, the influence of plumes of turbidity in front of estuaries in spring resulted in elevated average values of suspended particular matter (SPM) during this season with high variability. As expected, values of Chl a were higher in spring than in winter and autumn. Conversely, dissolved nutrients (NO_2_, NO_3_, PO_4_, SiOH_4_) were lower in spring compared with the other seasons (Supplementary Table S1). Niskin bottles were used to collect surface (1 m depth) water samples. Samples were immediately pre-filtered on a nylon-mesh filter of 200 µm to remove large zooplankton. The remaining fraction (< 200 µm) was then filtered through pre-combusted (450 °C for 4 h) GF/F filters until saturation (usually 1–2 L). Mesozooplankton was collected from vertical hauls (from 4 to 58 m depth) using a WPII net (200 µm mesh size). Fish larvae were caught using either a midwater ring net (winter and autumn) or a bongo net (spring) both with a mesh size of 500 µm. Fish larvae were sorted on board. All samples were immediately frozen at − 80 °C. All methods were carried out in accordance with relevant guidelines and regulation.

Stable isotope compositions (δ^13^C, δ^15^N values) from the main plankton species were used to decipher the primary energy pathways. In the laboratory, zooplankton and fish larvae were rinsed in distilled water, sorted, measured and identified to the lowest possible taxonomic level^46,47^. One to ~ 100 individuals of each taxon of similar size-classes were pooled together to ensure sufficient mass (~ 300 µg) for stable isotope analysis. Five to ten random individual measurements were taken from each pool to obtain average ranges of total lengths (mm). Samples were then freeze-dried, homogenized and ground to a fine powder. GF/F filters were observed under a stereomicroscope to remove nauplii and small zooplankton if present. Every filter was split in half and carbonates were removed from one half by fuming with HCl for subsequent δ^13^C analysis, the other half was kept for δ^15^N analysis. Filters were then freeze-dried to remove any excess water. Isotope ratios were measured with a Thermo Delta V isotope mass ratio spectrometer, coupled to a Carlo Erba NC 2500 elemental analyzer. The accuracy of the isotopic ratio measurements was checked by repeated analyses of an in-house standard (one analysis of the standard after every 10 samples) with an overall standard deviation of 0.2‰ for both elements. Stable isotopes ratios were expressed following the classical δ notation with:

$$\delta X = \left( {\frac{{R_{sample} }}{{R_{standard} }} - 1} \right) \times 10^{3}$$

where X being δ^13^C or δ^15^N, and R the isotopic ratios (13C/12C or 15N/14N, respectively) measured in samples and in international standards (Vienna Pee Dee Belemnite for C and atmospheric nitrogen for N).

In total, the sampling effort resulted in 552 measurements: 289 from zooplankton (18 taxa), 167 from fish larvae (13 taxa) and 96 from GF/F filters of water samples (Table 1).

**Table 1:** Number of measurements by species and by season (pools of 1 to 100 individuals). Mean and standard deviation (sd) of size (total body length, mm) and stable isotopes values for δ^15^N and δ^13^C. A total of 552 measurements of planktonic species are recorded.

	Species	Id_code	*n*	Mean size (mm)	sd size	Mean δ^13^C (‰)	sd δ^13^C	Mean δ^15^N (‰)	sd δ^15^N
Fish larvae									
Fall	Ammodytidae	Ammo	2	20.50	4.95	− 19.76	0.20	10.76	0.32
	Gobiidae	Gobbi	3	13.00	6.08	− 19.21	1.21	12.08	0.94
	*Pomatoschistus microps*	Pmicro	3	15.00	0.00	− 18.02	0.33	12.94	0.21
	***Sardina pilchardus***	**Spilc**	32	21.86	4.41	− 19.54	0.47	10.90	0.45
	*Syngnathus* spp.	Syngn	2	35.00	0.00	− 19.26	0.33	12.23	0.01
Spring	Ammodytidae	Ammo	5	14.80	1.48	− 19.78	0.94	9.21	0.32
	*Callionymus* spp.	Calli	11	5.05	1.17	− 19.52	0.65	11.40	0.69
	***Clupea harengus***	**Chare**	**29**	23.66	3.81	− 19.04	0.72	11.65	1.21
	Gobiidae	Gobbi	7	12.57	5.71	− 18.52	0.72	13.21	1.10
	***Limanda limanda***	**Llima**	**31**	10.19	2.62	− 19.31	0.71	12.56	0.51
	*Merlangius merlangus*	Mmerl	1	12.00		− 19.38		14.53	
	*Pholis gunnellus*	Pgunn	3	18.33	1.53	− 19.16	1.02	10.75	0.39
	*Sprattus sprattus*	Sspra	8	19.75	3.49	− 19.55	0.74	11.82	1.64
	*Trisopterus luscus*	Tlusc	2	10.25	6.72	− 21.01	2.97	12.16	2.13
Winter	*Ammodytidae*	Ammo	1	26.00		− 19.41		14.72	
	***Clupea harengus***	**Chare**	**14**	12.06	3.41	− 19.31	0.58	13.42	0.87
	***Pleuronectes platessa***	**Pplat**	**8**	8.85	1.12	− 21.68	0.51	14.91	0.49
	***Sardina pilchardus***	**Spilc**	**5**	21.60	1.95	− 19.10	0.57	13.74	1.91
Zooplankton									
Fall	*Acartia clausi*	Aclau	26	1.05	0.09	− 20.78	1.12	9.86	0.45
	Zoe Brachyura	Brach	5	2.24	0.66	− 17.88	1.09	9.16	1.18
	*Calanus helgolandicus*	Chaet	24	6.92	0.78	− 19.51	0.52	11.66	0.57
	*Centropages hamatus*	Chama	11	1.21	0.14	− 19.49	1.58	8.87	0.43
	**Chaetognatha**	**Chelg**	**7**	2.72	0.18	− 17.91	0.87	10.13	1.22
	Cumacea	Cuma	5	2.52	0.97	− 15.18	2.00	6.78	0.43
	*Ditrichocorycaeus anglicus*	Dangl	8	0.83	0.09	− 20.35	0.76	8.68	0.82
	*Euterpina acutifrons*	Eacut	8	0.61	0.04	− 19.31	0.85	7.93	1.07
	Gammaridae	Gamm	4	2.38	0.81	− 19.13	0.84	8.96	1.21
	*Labidocera wollastoni*	Lwoll	4	2.20	0.13	− 19.31	1.30	9.37	0.68
	Mysida	Mysi	4	7.68	3.30	− 18.38	1.04	10.21	0.96
	*Paracalanus/Pseudocalanus*	PaPse	22	0.79	0.05	− 20.48	0.81	8.58	0.69
	*Pisidia longicornis*	Plong	4	3.09	0.34	− 17.42	0.40	10.06	0.07
	*Temora longicornis*	Tlong	23	1.28	0.20	− 18.27	1.57	8.63	0.62
Spring	*Acartia clausi*	Aclau	9	1.37	0.05	− 21.25	0.89	7.27	0.35
	Zoe Brachyura	Brach	2	1.77	0.02	− 19.81	0.16	4.45	0.80
	*Calanus helgolandicus*	Chama	4	1.66	0.05	− 19.64	0.45	5.73	0.21
	*Centropages hamatus*	Chelg	3	2.58	0.00	− 19.44	0.58	7.38	0.49
	Cumacea	Cuma	1	3.10		− 18.39		4.88	
	Cypris	Cyprr	7	0.80	0.03	− 19.73	0.37	4.99	0.18
	*Euterpina acutifrons*	Eacut	5	0.64	0.00	− 19.83	0.97	2.03	0.51
	Mysida	Mysi	11	9.63	0.00	− 18.17	0.51	10.49	1.14
	Nauplii Cirripedia	Ncirr	7	0.45	0.02	− 21.71	0.72	3.77	0.57
	*Paracalanus/Pseudocalanus*	PaPse	7	1.19	0.14	− 20.73	0.33	6.07	0.24
	*Parapontella brevicornis*	Pbrev	3	1.75	0.08	− 20.37	0.28	6.43	0.13
	*Temora longicornis*	Tlong	19	1.54	0.18	− 19.48	0.36	6.58	0.57
Winter	*Acartia clausi*	Aclau	3	1.09	0.06	− 20.05	0.21	12.17	0.26
	*Crangon crangon*	Ccran	1	5.75		− 17.71		12.72	
	**Chaetognatha**	**Chaet**	**5**	7.93	1.19	− 19.20	0.47	12.84	0.47
	*Centropages hamatus*	Chama	1	1.46		− 18.79		10.54	
	*Calanus helgolandicus*	Chelg	8	2.61	0.23	− 19.82	0.59	9.15	1.94
	Cumacea	Cuma	3	2.19	0.51	− 16.21	0.56	6.92	0.75
	*Euterpina acutifrons*	Eacut	2	0.62	0.03	− 18.49	0.08	6.66	1.10
	Gammaridae	Gamm	2	2.47	0.83	− 19.12	0.12	9.32	0.85
	Mysida	Mysi	8	6.75	2.04	− 17.95	1.27	11.63	1.17
	*Paracalanus/Pseudocalanus*	PaPse	5	0.90	0.04	− 20.88	0.81	10.48	1.10
	*Temora longicornis*	Tlong	18	1.47	0.28	− 18.48	0.36	9.69	0.32
Seston									
Fall		POM	47			− 21.13	1.84	7.48	1.16
Spring		POM	18			− 20.58	1.23	5.21	0.75
Winter		POM	31			− 22.56	1.91	6.40	1.13

### Data analysis

#### Sources of variations in stable isotopes and size-structure models

One of the challenges when comparing food-webs over different spatial and temporal scales is the variation in baseline isotopes that needs to be accounted for when comparing consumers’ values. For this, we investigated the relationship between δ^13^C and δ^15^N isotopes and spatial and temporal factors, using a Generalized Additive Model (GAM) with a spatial smooth term. The GAM is a flexible statistical approach that allows for the modeling of non-linear relationships, making it well-suited for capturing complex patterns in environmental data. Prior to model fitting, we transformed the δ^13^C values to be positive (δ^13^C_positive_) by adding a constant value and then applied a log transformation (δ^13^C_log_) to meet the normality assumptions required for the analysis. The GAM models included a tensor product smooth of the “longitude” and “latitude” variables for *n* = 96 seston samples to account for potential spatial autocorrelation, and a categorical variable for « *Season*» with three levels (i.e. winter, spring or autumn) to include any seasonal effects on baseline stable isotopes values. Uneven seasonal sampling, with spring limited to 2017 and autumn/winter spanning multiple years (2018, 2019), hindered the complete capture of yearly influences on isotopic baselines. Before modeling, the “year” impact on winter and autumn baselines was explored. However, statistically non-significant effects prompted the pooling of data across years for a holistic model representation. The models were fitted using the ***gam*** function in the ***mgcv*** package in *R* with the Gaussian family and REML method for estimating the model parameters.

$$\begin{aligned} & GAM\_Carbon = \delta 13Clog \sim s\left( {longitude,latitude, bs = \text{``}tp\text{''},k = 30} \right) + Season \\ & GAM\_Nitrogen = \delta 15N \sim s\left( {longitude,latitude, bs = \text{``}tp\text{''},k = 10} \right) + Season \\ \end{aligned}$$

where *s(longitude,latitude, bs* =“ *tp”, k* = *10)* specifies a smooth term for the spatial coordinates, longitude and latitude, using a thin plate spline basis function with *k* degrees of freedom. The choice of *k* in our GAMs was pivotal to balance model complexity and effectively capture spatial variations in our isotopic data. In the carbon model, convergence challenges led to increasing knots to k = 30, addressing the intricacies in carbon isotope spatial variability without compromising model stability. Meanwhile, the nitrogen model used k = 10, striking a balance between capturing spatial patterns and maintaining model simplicity. The smooth term allows for flexible modeling of the spatial variation in the response variable. Convergence of the GAM models was assessed using several diagnostic plots (see Supplementary S1 for details). The predicted values, associated with particular coordinates and seasons, served as the isotopic baselines to adjust the observed isotopic data in zooplankton. This adjustment involved subtracting the predicted baselines from the observed zooplankton values. The resulting dataset, termed δ^13^C_adjusted_ or δ^15^N_adjusted_, reflects the refined values based on the derived isotopic baselines. Due to the log transformation in the carbon model, a back-transformation was required before this correction was applied for δ^13^C. Further details on spatial patterns on baseline estimates can be found in the Supplementary S1.

The three seasons (winter, spring and autumn) can then be compared and represented on a two-dimensional plane, the two axes of which being the adjusted isotopic signatures of nitrogen (δ^15^N_adjusted_) and carbon (δ^13^C_adjusted_). For each plot, a convex hull, illustrates the overall theoretical niche space occupied by the plankton and is the equivalent of the richness isotopic functional diversity metric proposed by Cucherousset and Villéger (2015). Details on formulas and calculations can be found in references^48,49^. The inside color polygons correspond to the niche space occupied by the plankton at each season. A larger seasonal polygon reflects a larger diversity of food resources and feeding strategies. At the species level, species close to the center of the seasonal polygon reflect more generalist species, and species at the edges reveal higher trophic divergence (i.e. specialized feeding preferences).

Linear mixed effect models (LMEM) were chosen to explore sources of variations (i.e. size, species, season) of stable isotope values for consumers. LMEM were particularly appropriate for the structure of our data, which encompass different grouping factors, unbalanced configuration with different sample sizes, and nested data (not truly independent data). All models were fit using the '*lmer*' function in the “***lme4****”*^50,51^ and “***vegan***^52^ packages in R version 4.1.0^51^, with restricted maximum likelihood (REML) estimation used to estimate the model parameters. To ensure model convergence and validate assumptions of normality and homoscedasticity of residuals, diagnostic plots of the fitted model were examined by using the package ‘***performanc*e**’^53^. Likelihood ratio tests via *anova()* were used to test the significance of both, fixed factors (i.e. season, size) and random effect structures by comparing full models against reduced models (see Supplementary S2 for details). Full models included the interaction between “Season” and “Size” (log transformed) as a fixed factors, with “(log_size | Species)” as a random effect thus accounting for potential interspecific variations within the size effect.

Best models for carbon and nitrogen were:

$$\begin{aligned} & modelC < - lmer(\delta 13C\;adjusted \sim Season * {\text{log}}\_size + (1 | Species)) \\ & modelN < - lmer(\delta 15N\;adjusted \sim Season * log\_size + (log\_size | Species) \\ \end{aligned}$$

### Energy fluxes

#### Food-web topology

A food-web topology was defined for the EEC from phytoplankton to fish larvae. Following the general methodology proposed by Planque et al. (2014)^54^, the topology consisted of three elements: nodes (i.e. species), links (i.e. trophic interactions), and directions (i.e. who is the predator and who is the prey). Trophic interactions and directions were constructed based on data from either peer-reviewed publications, gray literature or institutional reports (documented interactions, *TPlank0*). These were completed by inference, on the basis of knowledge on similar species from comparable regions and maximum prey/predator ratios (length of the largest prey divided by the length of the predator^55^ (potential interactions, *TPlank1*) (see Supplementary S3 for details). The presence of some groups in the plankton e.g. meroplankton, is limited to specific seasons or life stages on the basis of their specific traits, like fast growth or short life span. As a result, all species listed in the general topology do not necessarily meet and interact. Seasonal variations in plankton assemblages were investigated by constructing food-web topologies characteristic of winter, spring and autumn communities.

### Stable isotope mixing models

Isotope mixing models are based on the principle that a consumer’s isotope values result from the mixing of the isotope values of its food sources proportionally to their relative contributions to its diet (after adjustment for isotopic fractionation during digestion, metabolism, and assimilation, i.e. trophic enrichment factor TEF^56^). In this study, we used a combination of two mixing models (IsoWeb^57^ and MixSIAR^58^) to identify drivers of variation in energy pathways for main consumers as proposed by Giraldo et al. (2017)^59^ (see Supplementary S4 and S5 for details). MixSIAR is a consumer-scale mixing model and was used to explore variation (season, plankton size) in trophic pathways for predatory plankton. Prey sources were only considered if at least three samples were available. Permutational multivariate analysis of variance (PERMANOVA, 999 permutations)^60^ was used to test differences in centroids and dispersion (based on δ^13^C and δ^15^N values) among prey sources using the *adonis* function in the *vegan* package in R. Prior to permanova, the homogeneity of dispersion among the different species was tested using the *betadisper* function. Multilevel pairwise comparison (with Bonferroni-corrected p values) was then used to identify when two prey sources were indistinguishable based on their isotopic signatures (*pairwiseAdonis* package)^61^. Sources were aggregated a priori only if (i) there were no differences on their centroid position, (ii) the combined sources had some functional or ecological significance, and (iii), they were of similar sizes. Following suggestions by^62^, visual inspection of the final isotopic space (*i.e.* predator values should fall within the isotopic space created by prey values after TEF corrections) and correlation coefficients between isotopic values of prey sources were inspected. Species (or groups of species) with large negative correlation values (> 0.5) indicate that multiple solutions exists with either one of the species, which is reflected by larger credible intervals in the resulting posterior distribution. Seasonal variations of trophic interactions and isotope values were considered by running separate models for each season. To better visualize the importance of mesozooplankton prey according to their size-class, contributions where then aggregated a posteriori as a function of the maximum size of the species pools and corresponding to Seston (or POM), “ < 1 mm” for *Nauplii Cirripedia*, *Euterpina acutifrons* and Cypris; “1 to 1.5 mm” for *Acartia clausi*, *Paracalanus/Pseudocalanus* and *Ditrichocorycaeus anglicus; “*1.5 to 2 mm” for *Centropages hamatus*, *Parapontella brevicornis* and *Temora longicornis* and “ > 2 mm” for *Pisidia longicornis*, *Labidocera wollastoni*, Gammaridae, Cumacea, *Calanus helgolandicus* and Zoe Brachyura.

MixSIAR models were run using predator–prey seasonal TEF values (mean ± sd) previously calculated by IsoWeb (Supplementary S4). TEF variation across links was estimated, assuming that TEFs follow a normal distribution with a mean of 0.8 for carbon and 2.3 for nitrogen, as proposed for zooplankton food-webs^63,64^. Models were run seasonally with the following parameters: 106 chain length, 50 k burn-ins, and thin number 500 for three parallel Markov Chain Monte Carlo (MCMC) chains. Convergence was assessed using the Gelman-Rubin test (Gelman et al., 2014). MixSIAR models were run under the “very long” (10^6^ chain length, burn = 500.000, thin = 500, chains = 3) or “extreme” setting for complex model with a large number of prey (3*10^6^ chain length, burn = 1.500.000 thin = 500, chains = 3). Convergence was assessed using the default MixSIAR diagnostic Gelman-Rubin and Geweke tests (see Supplementary S5 for the complete posterior distribution outputs).

## Results

### Mesozooplankton food-web structure and functioning

#### Sources of variation of baseline SI values

Stable isotope values at the base of the planktonic food-web (*i.e.* seston) were highly variable and ranged from − 25.8‰ to − 16.2‰ for δ^13^C and from 3.4‰ to 9.5‰ for δ^15^N (Supplementary Fig. S1). Similarly, C:N ratios varied among seasons with lower values observed in spring (mean 9.13 ± 6.37) and higher values observed during autumn (12.4 ± 10.36) and winter (16.60 ± 12.53). GAM models for both nitrogen and carbon indicated that there was a non-linear relationship between the isotopic values and the spatial coordinates (*p* values < 0.001). In terms of the seasonal variability, both seasons, spring and winter were found to have significant negative effects on δ^15^N, indicating that δ^15^N tends to be lower in these seasons compared to autumn (*p* value < 0.01) (Table 2). Similarly, carbon values in winter were lower than in the other seasons. Further examination of the spatial patterns of estimated baseline values revealed that baseline estimates of δ^13^C are also significantly influenced by depth, while estimates of δ^15^N are explained by the interaction between depth and SPM, serving as a proxy for river influence (Supplementary S1).

**Table 2:** Summary of Generalized Additive Models (GAMs) on baseline isotopic values.

GAM_Nitrogen				
Smooth terms	edf	Ref.df	F	*p* value
S (longitude, latitude)	7.64	7.64	5.545	< 0.001 ***

Predictor	Value	Std. Error	t-value	*p* value
Intercept (i.e. autumn)	7.492	0.163	45.917	< 0.001***
Season (spring)	− 2.570	0.334	− 7.695	< 0.001***
Season (winter)	− 0.945	0.280	− 3.371	0.00113 **

GAM_Carbon				
Smooth terms	edf	Ref.df	F	*p* value
S (longitude, latitude)	13.18	13.18	3.19	< 0.001***

Predictor	Value	Std. Error	t-value	*p* value
Intercept (i.e. autumn)	1.763	0.061	28.56	< 0.001***
Season (spring)	0.034	0.126	− 0.275	0.784
Season (winter)	− 0.482	0.108	− 4.446	< 0.001***

The table presents smooth terms and parametric coefficients, along with estimated degrees of freedom (edf), reference degrees of freedom (Ref.df), F-values, and associated *p* values. For Nitrogen, the model explained 63.1% of the deviance with an R^2^ of 0.58, while for Carbon, the model explained 52.6% of the deviance with an R^2^ of 0.43.

#### Seasonal variations and size-structure

**Figure 2:**
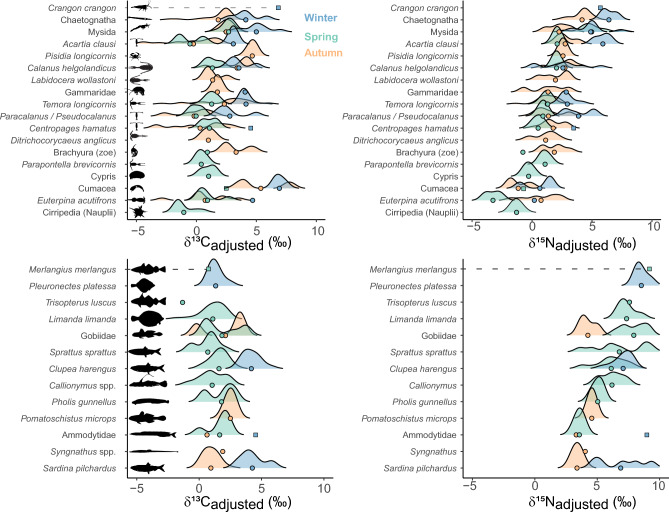
Variability (seasonal, inter, and intra-specific) of plankton isotopes (left, δ^13^C; right δ^15^N; top, mesozooplankton; bottom, fish larvae) in the EEC. Species are ordered based on their averaged δ^15^Nadjusted values. Seasonal mean values are illustrated by dots. Unique values (if n = 1) are illustrated by squares.

Among the 18 taxa of mesozooplankton identified in this study, eight were present during all seasons (*i.e*. *Acartia clausi, Calanus helgolandicus, Centropages hamatus,* cumaceans*, Euterpina acutifrons,* mysida*, Paracalanus sp. and Pseudocalanus sp.,* and* Temora longicornis*). Variations (seasonal, inter and intra-specific) of mesozooplankton isotope values (adjusted values corrected by spatio-temporal baseline variations) are shown in Fig. 2.

**Figure 3:**
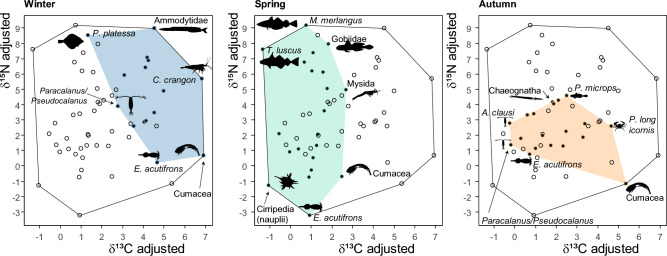
Seasonal variation of the isotopic niche space occupied by the plankton community in the EEC. The black polygon illustrates the overall theoretical isotopic niche space, which can be compared to the seasonal realized niches (in blue, green, and orange for winter, spring, and autumn, respectively). Seasonal data are represented as black dots, and species at the edges, reflecting those with a higher trophic divergence, are identified.

The comparison of the isotopic niche space occupied by the plankton community (Fig. 3) highlighted different planktonic food-web architectures. In winter, the isotopic niche space is positioned to the right (higher δ^13^C_adjusted_), reflecting an influence of benthic-derived carbon sources. Species with the highest isotopic divergence were cumaceans and *E. acutifrons* for zooplankton (lower end of δ^15^N_adjusted_), and fish larvae of *P. platessa* and Ammodytidae for higher δ^15^N_adjusted_ values. On the contrary, in spring, the isotopic niche space was centered around zero, thus reflecting a dominance of pelagic carbon-derived energy. Spring was also characterized by the higher range of δ^15^N_adjusted_ values, with minimum values for the copepod *E. acutifrons*, nauplii Cirripedia, and cumaceans, and maximum values for fish larvae *M. merlangius, T. luscus*, and Gobiidae, indicating a higher vertically structured community (i.e., a greater number of trophic levels). The smallest isotopic niche space was found in autumn, characterized by a relatively large range of δ^13^C_adjusted_ values, indicating the influence of both pelagic and benthic-derived carbon. Species displayed a more generalist strategy, as reflected by the lower range of δ^15^N_adjusted_ values.

**Figure 4:**
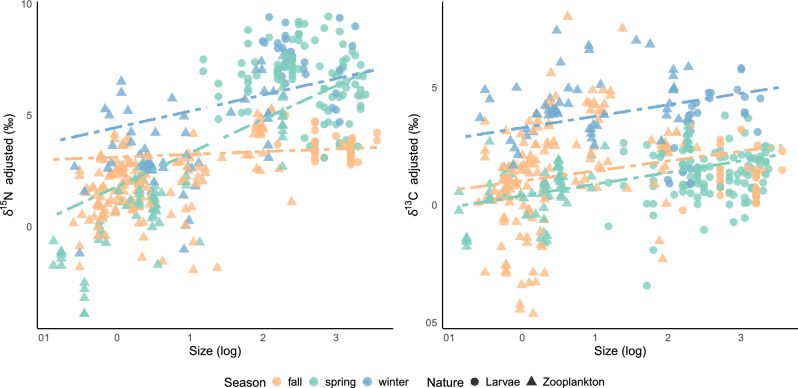
Seasonal and size effects on δ^15^Nadjusted and δ^13^Cadjusted values of the plankton community. Lines illustrate predictions from the Linear Mixed-Effects Models (LMEM) with 'Season' and 'Size' as fixed factors. The best carbon model includes 'Species' as a random effect, while the nitrogen model incorporates variation in size within species ('log_size | Species'). Zooplankton values are illustrated as triangles, and fish larvae are represented as circles, colored according to the season.

Linear Mixed Effect models in zooplankton indicated significant seasonal and size effects on both carbon and nitrogen isotope values (Table 3). For carbon, incorporating a random slope to account for size variation among species “(log_size | Species)” did not significantly improve the model fit compared to a model with only “Species” as a random intercept (See Supplementary S2). Values of carbon were lower in spring (estimate − 0.64) and higher in winter (estimate + 2.25). For nitrogen, there was a significant interaction between size and season. This indicates that the effect of size, represented by the slope, varies accross seasons, with a higher impact in spring compared to winter and autumn) (Fig. 4). The analysis of the random effect structure further indicated within size variations among species (see Supplementary S2).

**Table 3:** Linear Mixed Effect Models (LMEM) examining the influence of season, size, and species on zooplankton values. Marginal R^2^ and conditional R^2^ are used to assess the proportion of variance explained by fixed and random effects. σ^2^ represents unexplained variability in the response variable, while τ00 accounts for variability between species levels. *p* values are based on conditional F-tests using the Kenward–Roger approximation for degrees of freedom and the *pbkrtest*-R package.

Predictors	δ^13^Cadjusted				δ^15^Nadjusted			
	Estimates	CI	p	df	Estimates	CI	p	df
(Intercept)	1.03	0.34–1.73	**0.005**	43.62	3.12	1.68–4.57	** < 0.001**	25.38
Season [spring]	− 0.64	− 1.09 to − 0.18	**0.006**	449.99	− 1.32	− 1.72–− 0.93	** < 0.001**	375.93
Season [winter]	2.25	1.73–2.78	** < 0.001**	427.73	1.33	0.90–1.76	** < 0.001**	420.50
log size	0.42	0.04–0.80	**0.030**	91.01	0.12	− 0.41–0.66	0.646	25.77
Season [spring] × log size	0.07	− 0.24–0.39	0.648	428.48	1.39	1.08–1.69	** < 0.001**	88.92
Season [winter] × log size	0.07	− 0.27–0.40	0.696	446.18	0.61	0.33–0.89	** < 0.001**	434.51
Random effects								
σ^2^	1.57				1.05			
τ_00_	1.60_Species_				10.55_Species_			
τ_11_					0.80_Species.log_size_			
ρ_01_					− 0.97_Species_			
N	31_Species_				31_Species_			
Observations	456				456			
Marginal R^2^ /Conditional R^2^	0.300/0.653				0.241/0.884

### Food-web topology and energy fluxes

#### Size-structure seasonal variation of trophic pathways (MixSIAR models)

**Figure 5:**
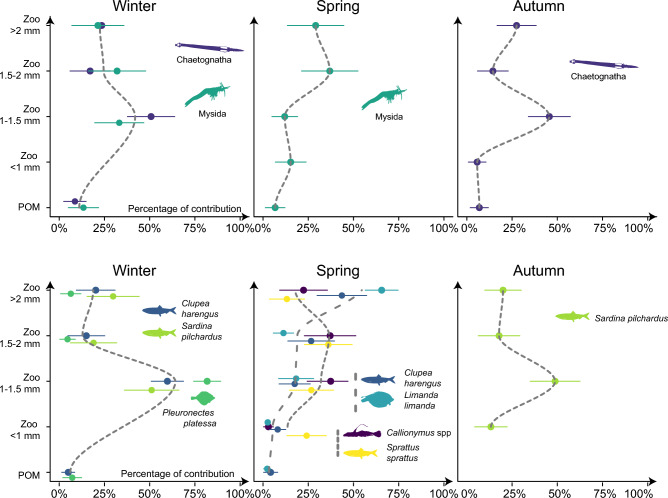
Diet composition (% diet) of plankton size-classes to predatory plankton. Values represent mean values and standard deviation posterior distributions of the MixSIAR models. Smooth dashed lines are for illustration purposes only and highlight main patterns or dominant size-classes to the diet.

Ichthyoplankton assemblages in **winter** were characterized by young-larval stages of herring (12 ± 3.4 SD mm SL) and plaice (8.85 ± 1.12 mm SL) and older stages of sardine (21.60 ± 1.95 mm SL). Among other carnivorous plankton, mysids and chaetognaths were also frequently encountered. Analysis of diet by size-classes showed that the main contributors for all winter species (representing between 51 to 82% of the diet) were between 1 to 1.5 mm in size corresponding to the copepods *A. clausi*, *Paracalanus*/*Pseudocalanus* spp, as well as *D. anglicus*. POM represented less than 10% of the diet for all species highlighting their predatory nature. Mysids diet was dominated by prey between 1 to 2 mm (both size classes accounting for ~ 66% of the diet) (Fig. 5). In **spring**, ichtyoplankton assemblages were characterized by older herring larvae (16 ± 3.8 mm SL). For herring, a clear pattern was found with a preference (44% ± 0.14 of the diet) for larger prey (> 2 mm) and a negligible contribution of POM that only accounted for 4% of the diet. A similar pattern was found for dab larvae (10 ± 2.6 mm SL), with a greater contribution of larger prey (66% ± 9% of the diet) and only negligible inputs from POM (~ 2%). For sprat larvae (20 ± 3.5% mm SL), there was an increased contribution from 24% for small zooplankton (< 1 mm) to 36% for medium-size zooplankton between 1.5 to 2 mm. Species > 2 mm contributed to 13% of the diet. *Callionymus* spp. Larvae diet was dominated by prey between 1 to 2 mm and mysids had a preference for prey > 1.5 mm. In **autumn**, Sardine larvae of similar sizes as those in winter were found (~ 21 mm SL). Contributions by size classes showed that main prey (49% ± 14% of the diet) were around 1 to 1.5 mm length. A similar patterns was found in Chaetognaths (~ 7–8 mm TL) that presented a pattern dominated by prey of intermediate sizes.

## Discussion

In this study, we investigated the variability in the planktonic food-web structure for a coastal ecosystem (the EEC), including mesozooplankton and fish larvae. We explored the isotopic niche space used by the plankton and its relationship with species’ size. Additionally, we employed stable isotopes mixing models to explore how different food-web architectures transferred up to higher trophic levels. Our results showed that food-web structure varied seasonally with size and highlighted different feeding patterns (trophic redundancy vs. trophic divergence) among seasons and species (Table 4). In the following sections, we discuss our results in light of (1) how the different structures and functions relate to changes in productivity and environmental drivers and (2) implications for the energy transfer to predatory plankton and in particular to fish larvae. Finally, (3) we discuss some of the remaining knowledge gaps and comment on what is needed to move forward.

**Table 4:** Summary of main findings, highlighting different food-web architecture indicators and their seasonal variation.

Food-web architecture	Indicator	Winter	Spring	Autumn
OM Source	δ^13^C Range, C:N Ratio	Influence of recycled organic matter/benthic origine	Dominance of pelagic-derived carbon sources (phytoplankton bloom)	Mix of benthic/recycled and pelagic organic matter (microbial-web)
Niche with and Community trophic structure	Total isotopic niche space & species overlap	Intermediate vertical structure and trophic divergence	Large. High vertical structure and trophic divergence	Small. High trophic redundancy
Vertical size-structure	Relationship (slope) of δ^15^N and size	Moderate	High	Low
Main prey-size for predatory plankton	Dominance of size classes	Low to intermedate size classes ~ 1 to 1.5 mm	Intermediate to high size classes > 1.5	Low to intermedate size classes ~ 1 to 1.5 mm

### Seasonal variation on planktonic food-webs

Following previous studies^19,21,65^, season emerged as a significant driver of variation for the mesozooplankton community structure. Overall, some of the variation could be attributed to seasonal differences in species composition contributing to the different size classes. However, a considerable number of species of similar sizes (~ 44%) were present throughout the year and exhibited substantial variation in their stable isotope composition, which indicates that the variability is also likely due to seasonal changes in species' diets. Flexibility of feeding strategies is a well-known and common pattern for copepods that are usually considered opportunistic omnivores. As an example, in spring, the planktonic harpacticoid copepod *E. acutifrons* was characterized by the lowest (and negative) values of δ^15^N_adjusted_, indicating that their isotopic composition was lower than the seston (proxy of POM) used as a baseline. This could be explained by the capacity of *E. acutifrons* to display selective feeding in ecosystems or instances with high suspended particulate matter levels^66^ (e.g., from increased rainfall or river discharge due to seasonal weather patterns) like those that characterize the EEC. On the contrary, nitrogen isotope values of *E. acutifrons* in winter and autumn were centered on zero, thus reflecting a more herbivorous diet when suspended particulate matter was low. Similar patterns were found for other copepods, such as *T. longicornis* (a suspension feeder)*,* or *A. clausi* (an ambush feeder). These species are considered mostly herbivorous but they can prey (and even preferentially select) heterotrophic protists (ciliates) when phytoplankton concentrations are low^31,67,68^. As expected, the highest δ^15^N_adjusted_ values were recorded in carnivore plankton (i.e., chaetognaths, mysids and fish larvae) for all seasons^21^, thus reflecting a higher trophic position for these species. However, the range of δ^15^N_adjusted_ for the autumn food-web was the lowest among all seasons (as reflected by the smaller isotopic niche space), and values for fish larvae were of the same order as some other mesozooplankton species, such as the shrimp *C. crangon* or the copepod *A. clausi*. A large range of δ^13^C_adjusted_ values was found in winter and autumn, indicating diversity in the origin of carbon sources (benthic and pelagic). Maximum values of δ^13^C_adjusted_ were found in cumaceans that had the highest trophic divergence at all seasons. Cumaceans feed mainly on microorganisms and organic material from the sediment, thus reflecting benthic organic matter consumption^69^. The presence of meroplanktonic larvae of benthic species further contributes to interactions between the plankton and the benthos^70^. Our results highlight the significance of benthic-pelagic coupling, a crucial pattern recognized in coastal ecosystems, especially in relatively shallow and well-mixed waters like the Eastern English Channel^59,71^. While previous studies focused on fish, our findings extend these vital connections to planktonic organisms, underlining the pervasive influence of benthic-pelagic coupling across multiple trophic levels.

**Figure 6:**
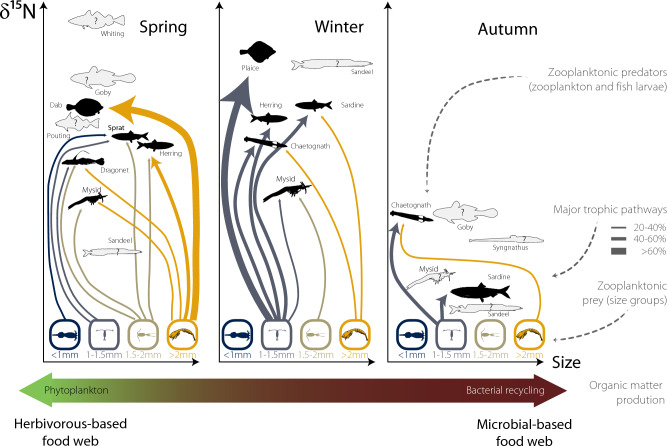
Schematic representation of the continuum of trophic structures for the EEC planktonic food-web.

Seasonal differences were also reflected in the size-structure of planktonic food-webs as observed by the different slopes and seasonal estimates of our LMEM models. Higher δ^15^N values with increasing size are frequently explained as reflecting size-related feeding patterns in marine plankton food-webs^23^. The relationship between nitrogen and size was strongest in spring, suggesting that the energy derived from phytoplankton blooms resulted in a more size-structured food-web and more specific predatory diets (higher trophic divergence as seen in the isotopic niche space). Similar trends have been found in tropical and subtropical regions where species tended towards more carnivorous feeding strategies leading to a higher vertical trophic structure (i.e., larger range of nitrogen isotope values or trophic levels), in periods of high Chl *a* (cold, non-stratified water) than during less productive seasons^24,72^. Additionally, strong trophic vertical structures during productive seasons have been hypothesized as the result of the accumulation of biomass and stronger microbial food-webs that increased food-chain length^19^. Conversely, the gentle slope of δ^15^N values with size in autumn, where consumers and prey displayed very similar values, suggests that the planktonic food-web is more dependent on recycled production^10^. These results are supported by the higher C:N ratios and more depleted δ^13^C values in autumn and winter, indicating a more processed material, possibly resulting from decomposition and recycling within the marine system and potentially from a benthic origin. Recent studies have shown that at least some protists may exhibit variations in ^15^N trophic enrichment that deviate from the well-established patterns observed in metazoan consumers. Therefore, it is possible that when microbial activity dominates the energy pathways in the plankton, it leads to lower δ^15^N values for consumers^26^. Conversely, lower C:N ratio and less depleted δ^13^C values observed in spring suggest a period of increased primary productivity, likely due to phytoplankton blooms. Linked to the isotopic niche space occupied in each season, trophic diversity was highest in spring and lowest in autumn (generalist species—trophic and functional redundancy). Our results are in agreement with previous studies on the Mediterranean suggesting that food overlap (or trophic redundancy) among zooplankton species and size classes seems higher during less productive (summer-autumn) than during the high productivity seasons (late winter-spring)^65^. In line with recent observations for the north Atlantic, our data highlights that zooplankton food-webs are organized in complex trophic structures that are not easily summarized into 2–3 functional groups disregarding seasonal inter- and intraspecies variation in feeding patterns. The seasonal variations in the planktonic food-web architecture from spring to winter and autumn align with previous observations of a continuum of trophic structures where 'herbivorous-based food-webs' vs 'microbial-based food-webs' represent only extreme configurations of the transient nature of a single planktonic food web. These configurations vary seasonally, depending on nutrient availability, phytoplankton production, and bacterial activity^73^ (Fig. 6).

### Energy transfer to predatory plankton

Fish larvae are key players in food-web and population dynamics. Their survival is considered one of the main processes influencing stock recruitment variability^1,2^ and is closely linked to their capacity to capture suitable prey^3,74,75^. Considered mainly omnivorous/carnivorous feeders, research over the past decades has shown that fish larvae can display different feeding strategies in order to reduce potential interspecific competition with other predators^76,77^. Similarly, ontogenetic changes linked with body size (and by extension mouth size) suggest that most species switch to bigger prey as they grow^78^. However, prey selection based on species-size, and trade-offs between prey availability and capturability are also part of fish larvae foraging strategies^32^. In this study, we investigated possible carry-over effects of the different seasonal planktonic food-webs structures on fish larvae and other carnivorous plankton.

In winter, the ichthyoplankton assemblage of the EEC is dominated by herring larvae after spawning of the Downs herring component. Plaice and sardine larvae are also frequently encountered, although to a lesser extent. The former species have been reported to be omnivorous at their larval stage based on stomach content analysis^30,79,80^, and variation of diet among regions has led researchers to believe that they might feed on the most abundant prey^81,82^. The use of stable isotope mixing models is complementary to stomach contents analyses, as the former reflects the assimilated diet while the latter informs on ingested diet. Our results agree with previous observations indicating that winter predators (including mysids and chaetognaths) can feed on a wide variety of prey sources. Theoretically, under food-limited conditions, fish larvae cannot afford to select prey and should ingest a wider range of prey sizes^83^. However, experimental studies show that at colder temperatures (as expected in winter during low production) larvae depend more heavily on optimal prey sizes^32^. This is in agreement with stable isotopes mixing models indicating that small copepods (~ 1 to 1.5 mm) dominate the diet of winter predators. Unfortunately, we had no data on zooplankton abundance by size-classes to test if small copepods correspond to the most abundant size-class, thus supporting that fish larvae in winter behave as opportunistic predators. Similarly, the contribution of phytoplankton to fish larvae has been reported as an important food for first-feeding and young larvae that can use diatoms as a type of initial or exploratory food source, to establish their feeding behavior^82,83^. In our study, seston (POM) appeared as a negligible contributor to the diet (< 10% of the diet) suggesting that older larvae are more carnivorous than omnivorous and that the nutritional value of POM is rather limited. The surprisingly large contribution (> 75%) of small copepods for plaice larvae might however, be an overestimation, as data on one of the main potential prey of plaice, the appendicularian *Oikopleura dioica*^81^ was missing (see below discussion on missing species)*.* However, previous studies have shown that small copepods such as *A. clausi* and *Para-Pseudocalanus* have poor escape capabilities^84^ that might lead to a positive selection by fish larvae^78^.

Ichthyoplankton in spring was more diverse when compared to the other seasons, and so we expected some differences in feeding patterns among fish larvae to lower possible interspecific competition. Following the strong size-structure of the zooplankton food-web, dietary patterns in herring and dab larvae indicate a dominance of bigger prey in the diet (> 1.5 mm) and underline the importance of large copepods such as *C. helgolandicus* for the transfer of energy to higher trophic levels. Herring larvae in spring are bigger when compared to individuals collected in winter and are expected to be more successful at capturing bigger prey. Still, larger prey also dominated the diet of the dab larvae (~ 10 mm). Contrary to previous gut content analysis that found that dab feeds mainly on small items (nauplii and copepodites of *T. longicornis*)^85^, our results suggest that small prey represent less than 10% of the assimilated diet in the EEC. Sprat and dragonet larvae did not seem to have a preferred or dominant size-class. Results for sprat are in agreement with previous studies that show that trophic niche breadth increased with larval’ size from newly hatched to pre-schooling larvae (~ 16 mm) but then remained unchanged with sprat larvae feeding (and selecting) prey of different size-classes such as *Acartia* spp and *C. hamatus*^83^. The contribution of larger prey such as *C. helgolandicus* only represented ~ 13 to 24% of the diet, which suggest that some predator species might have adapted their feeding strategy to a more generalist diet to lower possible inter-specific competition and avoid trophic niche overlap. In agreement with previous studies, mysids appeared as carnivorous, feeding on similar zooplankton prey as fish larvae. The only exception was the negligible contribution of *A. clausi* (~ 5%, see Supplementary S5) that seems to be rejected by mysids even when the prey is abundant in the water^86,87^.

In autumn, larvae of several fish species were recorded but unfortunately only sardine larvae were collected in sufficient numbers for stable isotopes mixing models. Transfer of energy to predatory plankton was therefore only explored for sardine and chaetognaths that were also frequently encountered. Both predators fed on a variety of species from different size-classes. Contrary to herring, sardine larvae have a late spring–summer and autumn spawning season^88^ so that larvae collected at both periods were of similar sizes. Similar to the winter pattern, small prey (around 1 to 1.5 mm) dominated the diet of sardine larvae. In autumn, contrary to other seasons, δ^15^N_adjusted_ values for chaetognaths and mysids were similar to those of fish larvae, suggesting that both groups share a similar trophic level and might feed on similar resources. These results concurs with the smaller isotopic niche space and higher trophic redundancy for the plankton food-web observed during autumn.

Overall, seasonal patterns of planktonic food-webs seem to propagate to upper trophic levels including fish, in particular at low productive seasons. A recent study^89^, looking into the plasticity of adult fish assemblages in the EEC during autumn and winter, also showed a reduction of the isotopic niche space and number of trophic levels in autumn, and a higher vertical structure in winter. The authors suggested that changes in feeding strategies were probably the result of differences on primary production (leading to changes in prey abundances and possible competition or niche overlap). Although we have no data on zooplankton abundance to confirm this observation, foraging on similar prey in autumn suggests that there is no clear density dependence, which leads to trophic similarity^90^. In spring, different feeding patterns related to prey size suggest that other factors than consumer’ body size (e.g., resource partitioning or competition) influence larval feeding strategies during productive periods.

### Remaining knowledge gaps and future directions

Other sources of variation: There are multiple sources of variation and uncertainties when using stable isotopes to elucidate trophic patterns^91–93^. For instance, possible inter-annual variations on plankton stable isotopes were not explored in this study because of data limitations. However, studies have shown that baseline values can vary spatially (isoscapes) but that these spatial patterns are stable from year to year (summer values over 10 years in the North Sea)^94^. Additionally, recent analysis of zooplankton community and size structure between 1991 to 2013 in the EEC in winter, showed that patterns (in terms of community composition, abundance and size-structure) where relatively stable over time within the region of our study^42^. Spatial and environmental patterns can also influence isotopic values at the base of the food-web. The distinct spatial trends in seston δ^13^C and δ^15^N are likely a result of a complex interplay of factors, including resource availability for phytoplankton, phytoplankton community structure, and the mixing of organic matter from various sources. These factors exhibit strong spatial gradients from the coast to offshore and/or from the distance to the river plume^95–97^. This clearly paves the way for further investigation into the factors influencing baseline reference values. Notably, our results align with isoscapes estimated using alternative modeling approaches (i.e. integrated nested Laplace approximation, INLA), as applied in a study of predatory gelatinous zooplankton in the EEC during 2015–2016^97^. In that context, temperature, phytoplankton taxonomy, terrestrial nutrient input (at a relatively local scale), and mixing degree emerged as pivotal factors shaping isotopic baseline structures^98^. Taken together, these studies suggest that major changes in both zooplankton communities and stable isotopes in the EEC are likely driven by changes in temperature (probably an indirect link) and productivity, supporting our statement that season is the main driver of variability for planktonic food-webs at relatively low spatial scales.

Missing species: Copepods represent the majority (~ 90%) of the mesozooplankton in the EEC^42^. However, gelatinous zooplankton (Cnidaria, Ctenophora, Tunicata) are also frequently encountered^99,100^ and can occasionally occur in large numbers with biomass exceeding that of fish in oligotrophic waters^21^. Unfortunately, samples of gelatinous zooplankton were not preserved from our surveys and so knowledge on their trophic dynamics and seasonal variability for the EEC planktonic food-web remains limited. Even though some species are too fragile and difficult to identify, samples of large cnidarians and ctenophores can help elucidate the trophic structure of gelatinous species and possible predation or niche overlap with fish larvae^101^. Joint information on crustaceans, gelatinous zooplankton and ichthyoplankton can be used as indicators of energy flow and trophic pathways, which should inform on how planktonic communities respond to environmental changes. Such information is required to inform several management descriptors (e.g. OSPAR indicators, Marine Strategy Framework Directive-D1—Biological Diversity D4—Marine Food-webs, https://oap.ospar.org/).

## Conclusion

In conclusion, our study highlights significant seasonal variability in the planktonic food-web in the Eastern English Channel and Southern Bight of the North Sea. These dynamics, intricately linked to fluctuations of suspended particulate organic matter and primary production, give rise to distinct variations in isotopic niche spaces and trophic structure (both vertically in terms of trophic levels, and horizontally in terms of carbon source variability), as well as size-structural patterns. Remarkably, the food-web architecture of lower trophic levels propagate upwards to carnivorous plankton including fish larvae, and even higher up to adult fish. This emphasizes the pivotal role of bottom-up control in shaping coastal systems like the EEC, and the need for regular, long-term monitoring of lower trophic levels across the full seasonal and spatial gradients of any given management area. A better understanding on how environmental parameters shape trophic transfers is essential to predict how planktonic food-webs will respond to global change scenarios. Furthermore, the intrinsic trophic link between mesoplankton and fish larvae (alongside other vital resources for sustaining human consumption) underscores the necessity for enhanced management and an ecosystem-based approach that includes planktonic species based on life-history traits and size spectra.

### Supplementary Information

Supplementary Information.

## Data Availability

The datasets generated during and/or analysed during the current study are available from the corresponding author on reasonable request.
