# Supplementary material for: Insights into planktonic food-web dynamics through the lens of size and season
Source: Sci Rep. 2024 Jan 19;14:1684. doi: 10.1038/s41598-024-52256-4 (PMC10798955; doi:10.1038/s41598-024-52256-4)
Supplement: Supplementary file 1 — Supplementary Information. [file 41598_2024_52256_MOESM1_ESM.docx]

**Supplementary information for :**

**Insights into planktonic food-web dynamics through the lens of size and season**

Carolina Giraldo^1^*, Pierre Cresson^1^, Kirsteen MacKenzie^1^, Virginie Fontaine^1,2,^ Christophe Loots^1^, Alice Delegrange^2^ & Sébastien Lefebvre^2^.

# **Supplementary Section S1**

# **« Sources of variation of baseline isotopic values using Generalized Additive Models (GAM) and environmental drivers »**

**Seasonal variation seston and environmental drivers:**


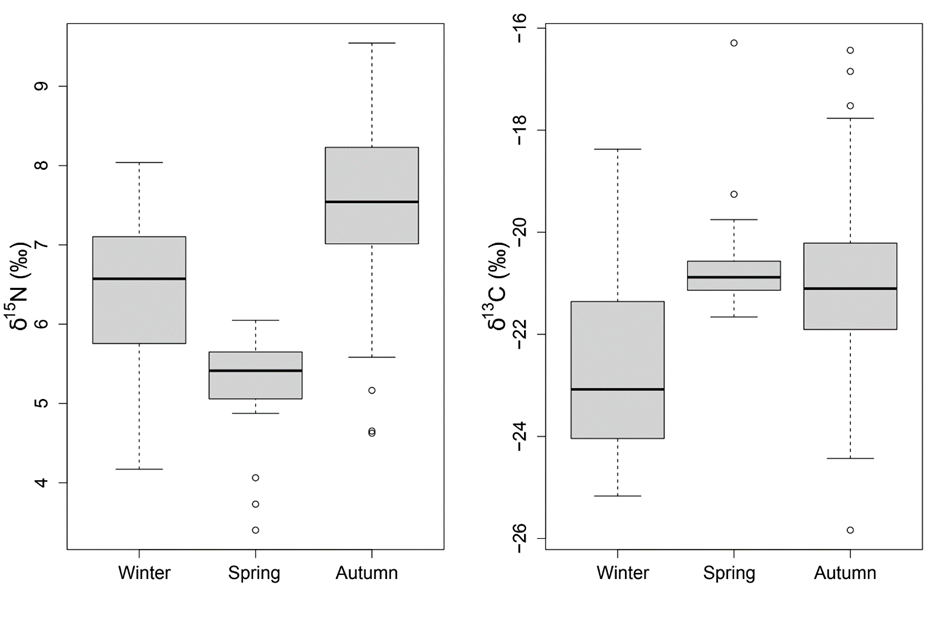


**Figure S1 : Seasonal variation of stable isotopes values of seston.**

**Table S1: Seasonality of environmental parameters.** Mean (± sd) averaged seasonal values between 2017 and 2019.

| **Parameter** | **Winter**  (Jan – Feb) | **Spring**  (March – May) | **Autumn**  (Sep – Oct) |
| --- | --- | --- | --- |
| Temperature (°C) | 8.59 ± 0.7 | 11.27 ± 2.6 | 16.96 ± 0.4 |
| Salinity | 34.43 ± 0.6 | 34.52 ± 0.2 | 34.14 ± 0.5 |
| SPM (µg L^-1^) | 9.37 ± 11.9 | 44.93 ± 33.9 | 12.04 ± 6.8 |
| Chl *a* (µmol L^-1^) | 1.15 ± 2.2 | 7.43 ± 4.8 | 2.56 ± 1.8 |
| NO_2_ (µmol L^-1^) | 0.56 ± 0.3 | 0.27 ± 0.2 | 0.29 ± 0.2 |
| NO_3_  (µmol L^-1^) | 11.20 ± 5.1 | 5.09 ± 3.7 | 10.05 ± 7.4 |
| PO_4_ (µmol L^-1^) | 0.54 ± 0.1 | 0.15 ± 0.1 | 0.26 ± 0.2 |
| SiOH_4_ (µmol L^-1^) | 6.69 ± 3.7 | 1.82 ± 1.4 | 6.95 ± 4.3 |

**Convergence and diagonstic plots for model performance :**

To ensure the validity of our GAM models, we checked for convergence using several diagnostic plots. First, we examined the standardized residuals versus fitted values plot of the model using the ***plot()*** function in R. This plot presents four different perspectives on model residuals through four distinct panels. In summary, the plot function showed that the residuals were well-behaved and centered around zero, indicating that the model adequately captured the variability in the data (Supplementary Fig. S3 and S5). Next, we used the ***vis.gam()*** function to generate an contour plot of the GAM, which allowed us to visualize the overall spatial patterns in the data (Supplementary Fig. S2 and S4). Finally, we used the ***gam.check()*** and ***concurvity()*** functions to check for convergence and potential issues with collinearity among the model terms. The gam.check function conducted a statistical tests for the spatial smooth and 'k' values. In this context, a small p-value suggests that residuals are not randomly distributed and that there aren't enough basis functions. In such cases, the number of 'k' should be increased, and the test should be repeated. In our case, these diagnostic tests indicated that the models were well-specified and that the model terms were not overly correlated, ensuring the validity of our results.


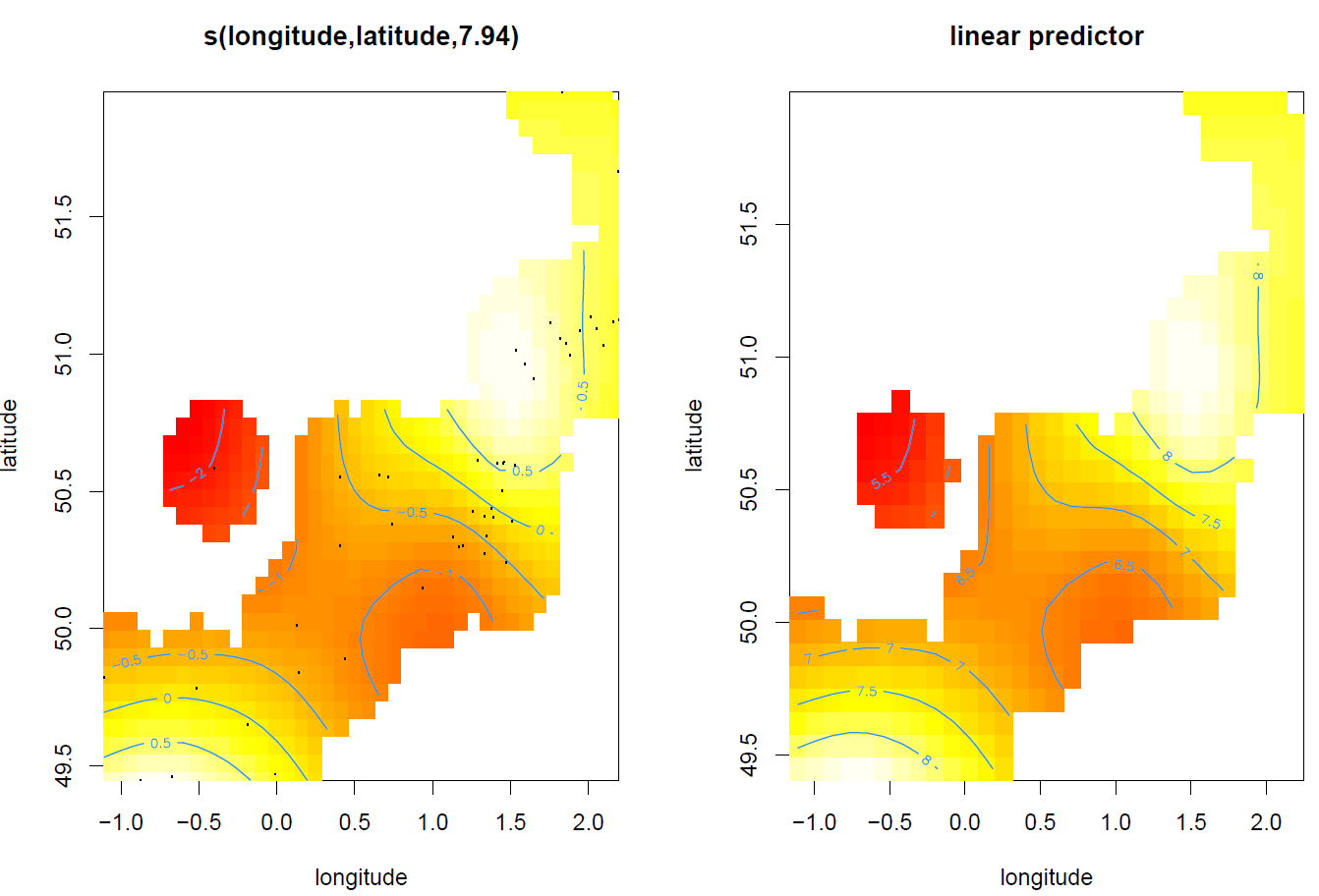


**Figure S2: Contour plot and overall spatial patterns of seston δ^15^N from the GAM modelling.** The term’s (longitude, latitude)' in the first plot indicates the smooth term for latitude and longitude, revealing the spatial pattern captured by the model. Deviations in color (yellow to red) highlight areas of negative (red) or positive (yellow) effects. The second plot on the right focuses specifically on the spatial distribution of model predictions. For illustration purposes we allowed extrapolation as far as 10% of the actual data.


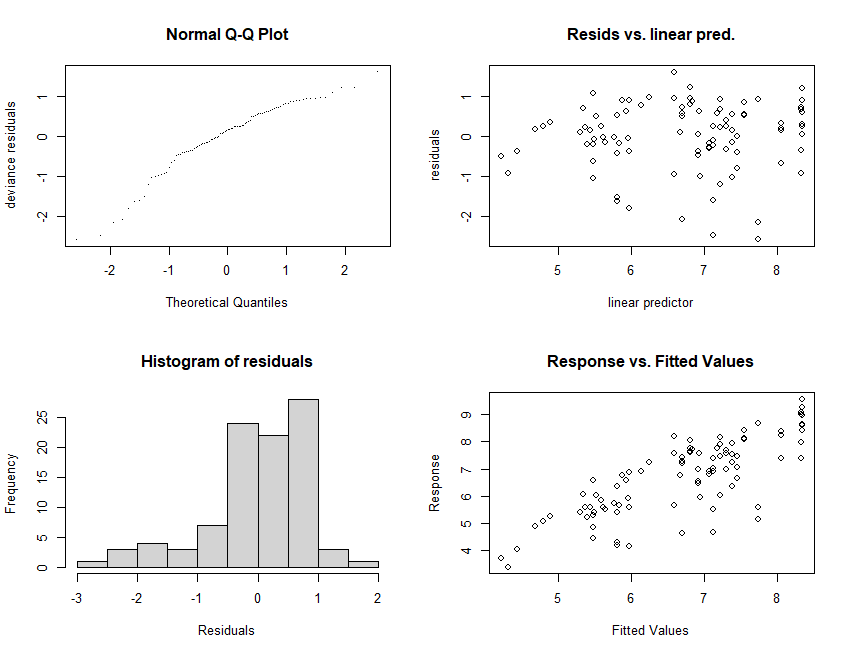


**Figure S3: Convergence plots for GAM on δ^15^N.** The first panel showcases a Q-Q plot, comparing model residuals to a normal distribution. A well-fitted model will exhibit residuals that closely align with a straight line. In the bottom left panel, a histogram of residuals is presented, expected to have a symmetrical bell shape. The top-right panel displays residuals values, which ideally should be evenly distributed around zero. The final panel in the bottom right shows the response versus fitted values, where a perfect model would yield a straight line.


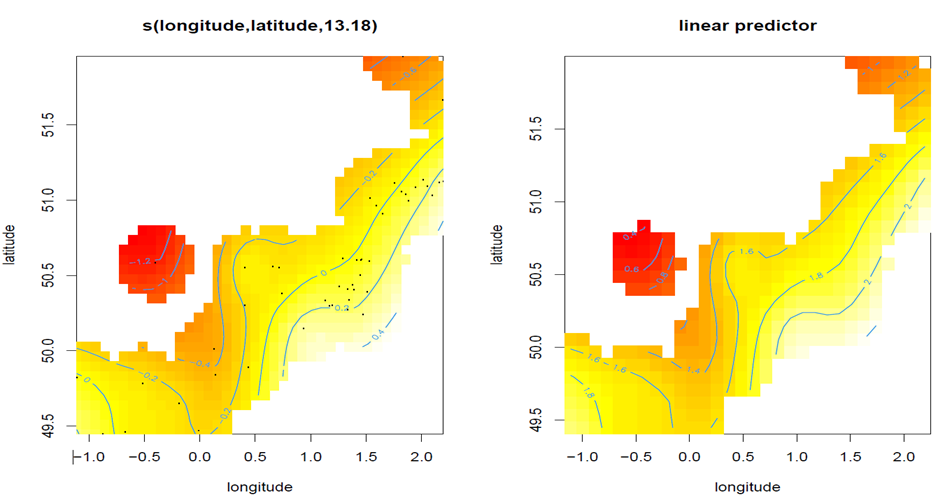


**Figure S4: Contour plots and overall spatial patterns of seston transformed δ^13^C values (positive and log transformed) from the GAM modelling.** The term’s (longitude, latitude)' in the first plot indicates the smooth term for latitude and longitude, revealing the spatial pattern captured by the model. Deviations in color (yellow to red) highlight areas of negative (red) or positive (yellow) effects. The second plot on the right focuses specifically on the spatial distribution of model predictions. For illustration purposes we allowed extrapolation as far as 10% of the actual data.

A representation of the spatial distribution of absolutes residuals to emphasize the magnitude of the deviations from the model predictions is illustrated on Supplementary Fig. S5 and S7. :


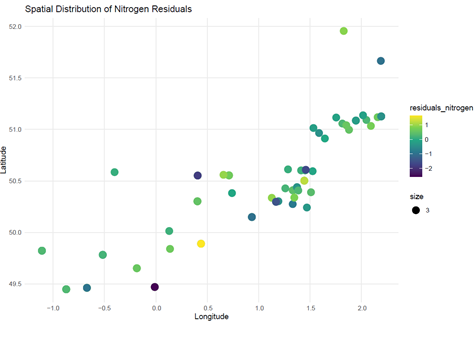


**Figure S5: Visual representation of the spatial distribution of absolute residuals**. The graph illustrates the magnitude of deviations from the model predictions. Each point on the map corresponds to a specific location, with color intensity indicating the magnitude of the residuals. More intense colors represent greater deviations.


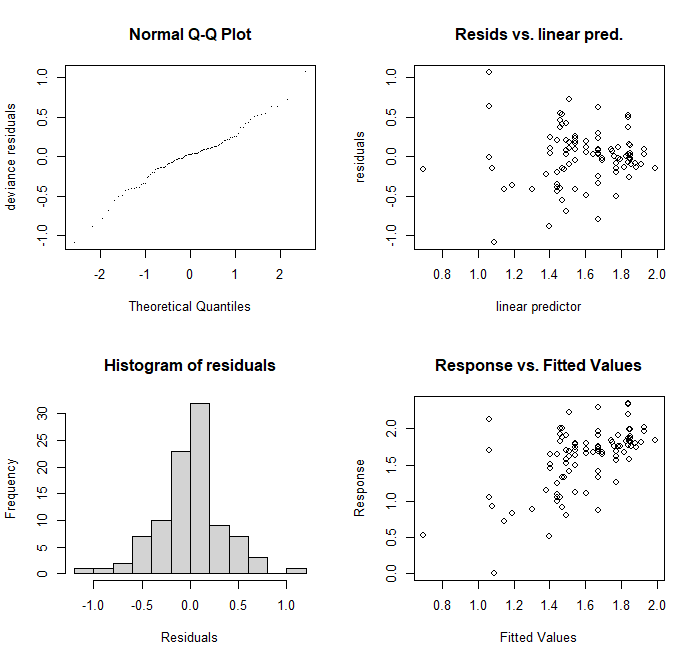


**Figure S6: Convergence plots for GAM on δ^13^C.** The first panel showcases a Q-Q plot, comparing model residuals to a normal distribution. A well-fitted model will exhibit residuals that closely align with a straight line. In the bottom left panel, a histogram of residuals is presented, expected to have a symmetrical bell shape. The top-right panel displays residuals values, which ideally should be evenly distributed around zero. The final panel in the bottom right shows the response versus fitted values, where a perfect model would yield a straight line.


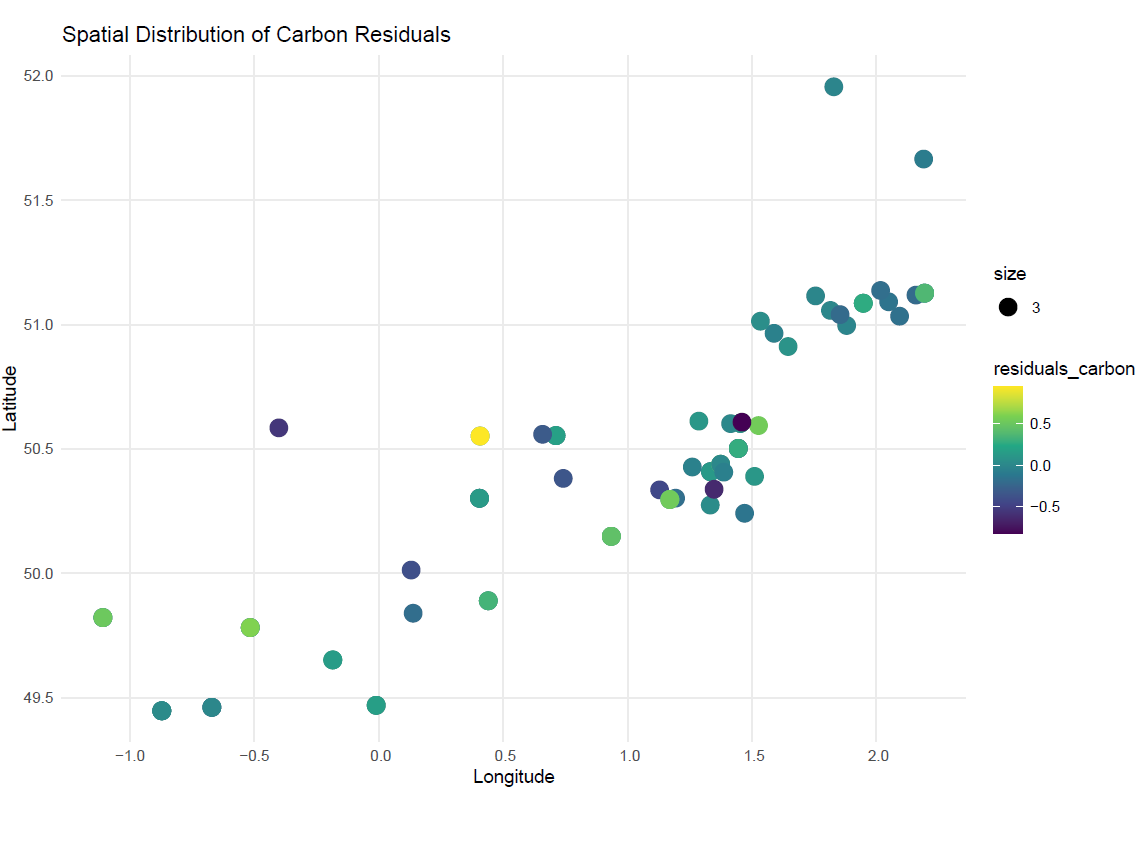


**Figure S 7: Visual representation of the spatial distribution of absolute residuals**. The graph illustrates the magnitude of deviations from the model predictions. Each point on the map corresponds to a specific location, with color intensity indicating the magnitude of the residuals. More intense colors represent greater deviations.

Spatial patterns from estimated values was further explored using a Generalized Linear Model. Our additional analyses revealed that baseline estimates of δ^13^C are significantly influenced by depth, while baseline estimates of δ^15^N are explained by the interaction between depth and Suspended Particulate Matter (SPM), serving as a proxy for river influence.

Outputs of the GLM for Nitrogen

glm(formula = δ^15^Npred ~ Depth * log(MEStotale), family = gaussian(link = "log"), data = data)

Coefficients:

Estimate Std. Error t value Pr(>|t|)

(Intercept) 2.540673 0.110526 22.987 < 2e-16 ***

Depth -0.017766 0.003534 -5.027 2.77e-06 ***

log(SPM) -0.267703 0.040147 -6.668 2.58e-09 ***

Depth:log(SPM) 0.007713 0.001358 5.678 1.90e-07 ***

---

Signif. codes: 0 ‘***’ 0.001 ‘**’ 0.01 ‘*’ 0.05 ‘.’ 0.1 ‘ ’ 1

(Dispersion parameter for gaussian family taken to be 0.7208045)

Null deviance: 101.685 on 87 degrees of freedom

Residual deviance: 60.547 on 84 degrees of freedom

AIC: 226.83

Outputs from the GLM for Carbon

glm(formula = δ^13^Cpred ~ Depth * MES_totale, family = gaussian(link = "log"), data = data)

Coefficients:

Estimate Std. Error t value Pr(>|t|)

(Intercept) 6.116e-01 5.512e-02 11.095 < 2e-16 ***

Depth -4.824e-03 1.830e-03 -2.636 0.00998 **

SPM 1.417e-03 1.645e-03 0.861 0.39162

Depth: SPM -4.948e-05 9.079e-05 -0.545 0.58721

---

Signif. codes: 0 ‘***’ 0.001 ‘**’ 0.01 ‘*’ 0.05 ‘.’ 0.1 ‘ ’ 1

(Dispersion parameter for gaussian family taken to be 0.04822654)

Null deviance: 5.3541 on 87 degrees of freedom

Residual deviance: 4.0510 on 84 degrees of freedom

AIC: -11.163

## Prediction for baseline corrected values and environmental drivers:

The ***predict.gam*** function was used to predict baseline values of δ^13^C and δ^15^N for every sampling station and season from which zooplankton was collected.

The predicted values of carbon where then back-transformed :

$$d13C\_pred\_back <- exp(d13C\_predicted) - 1 + min(d13Cphyto)$$

Adjusted zooplankton values were then calculated as follows :

$$Zooplankton\_d13Cadjusted <- d13Czoo - d13C\_pred\_back$$

$$Zooplankton_{d15Nadjusted}<- d15Nzoo - d15Npredicted$$

Where $d13C\_pred\_back$ and $d15Npredicted$ corresponds to the baseline phytoplankton value predicted by the GAMs models (Fig. S8).


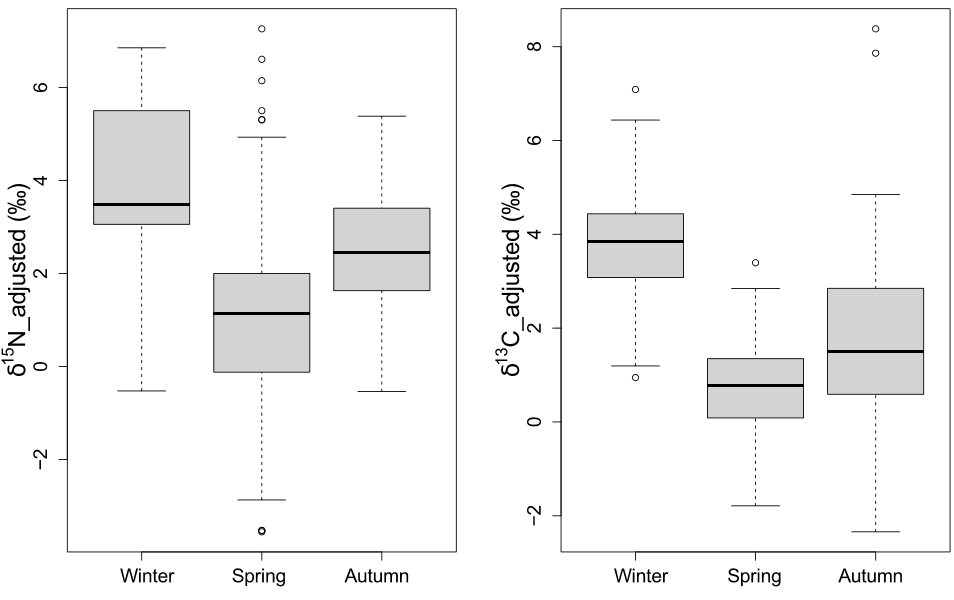


**Figure S 8: Seasonal variation of zooplankton.** Values of both nitrogen and carbon were adjusted to take into account baseline seasonal variations and allow for a direct comparison among different seasons.

# **Supplementary Section S2**

# **« Linear Mixed Effect Models on the effect of season and size on zooplankton isotopic values »**

Model selection and comparison of different linear mixed-effects models for δ^15^Nadjusted and δ^13^Cadjusted values. Tables include the number of parameters (npar), Akaike Information Criterion (AIC), Bayesian Information Criterion (BIC), log-likelihood (logLik), deviance, Chi-square statistic (Chisq), degrees of freedom (Df), and the p-value for the Chi-square test (Pr(>Chisq)).

# Nitrogen :

Test of random effect structures on the full model (best model in bold):

Models:

nestedN1: δ^15^N_adjusted_ ~ Season * log_size + (1 | Species)

**nestedN2: δ^15^N_adjusted_ ~ Season * log_size + (log_size | Species)**

npar AIC BIC logLik deviance Chisq Df Pr(>Chisq)

nestedN1 8 1446.5 1479.5 -715.25 1430.5

nestedN2 10 1441.4 1482.7 -710.72 1421.4 9.0706 2 0.01072 *

---

Signif. codes: 0 ‘***’ 0.001 ‘**’ 0.01 ‘*’ 0.05 ‘.’ 0.1 ‘ ’ 1

Test of significance of fixed factors (best model in bold):

Models:

nestedN3: δ^15^N_adjusted_ ~ log_size + (log_size | Species)

nestedN4: δ^15^N_adjusted_ ~ Season + (log_size | Species)

**nestedN2: δ^15^N_adjusted_ ~ Season * log_size + (log_size | Species)**

npar AIC BIC logLik deviance Chisq Df Pr(>Chisq)

nestedN3 6 1629.1 1653.8 -808.56 1617.1

nestedN4 7 1524.1 1553.0 -755.05 1510.1 107.023 1 < 2.2e-16 ***

nestedN2 10 1441.4 1482.7 -710.72 1421.4 88.661 3 < 2.2e-16 ***

---

Signif. codes: 0 ‘***’ 0.001 ‘**’ 0.01 ‘*’ 0.05 ‘.’ 0.1 ‘ ’ 1

# Carbon:

Test of random effect structures on the full model (best model in bold):

# Models:

# **nestedC1: δ^13^Cadjusted ~ Season * log_size + (1 | Species)**

# nestedC2: δ^13^Cadjusted ~ Season * log_size + (log_size | Species)

# npar AIC BIC logLik deviance Chisq Df Pr(>Chisq)

# nestedC1 8 1581.5 1614.5 -782.74 1565.5

# nestedC2 10 1585.3 1626.5 -782.66 1565.3 0.1454 2 0.9299

Test of significance of fixed factors :

Models:

nestedC3: δ^13^Cadjusted ~ log_size + (1 | Species)

nestedC4: δ^13^Cadjusted ~ Season + (1 | Species)

**nestedC1: δ^13^Cadjusted ~ Season * log_size + (1 | Species)**

npar AIC BIC logLik deviance Chisq Df Pr(>Chisq)

nestedC3 4 1766.6 1783.1 -879.29 1758.6

nestedC4 5 1583.7 1604.3 -786.85 1573.7 184.8708 1 < 2e-16 ***

nestedC1 8 1581.5 1614.5 -782.74 1565.5 8.2355 3 0.04139 *

---

Signif. codes: 0 ‘***’ 0.001 ‘**’ 0.01 ‘*’ 0.05 ‘.’ 0.1 ‘ ’ 1

Model quality was assed by using the ’performance’ package (Supplementary Fig. S11 & Fig. S12).


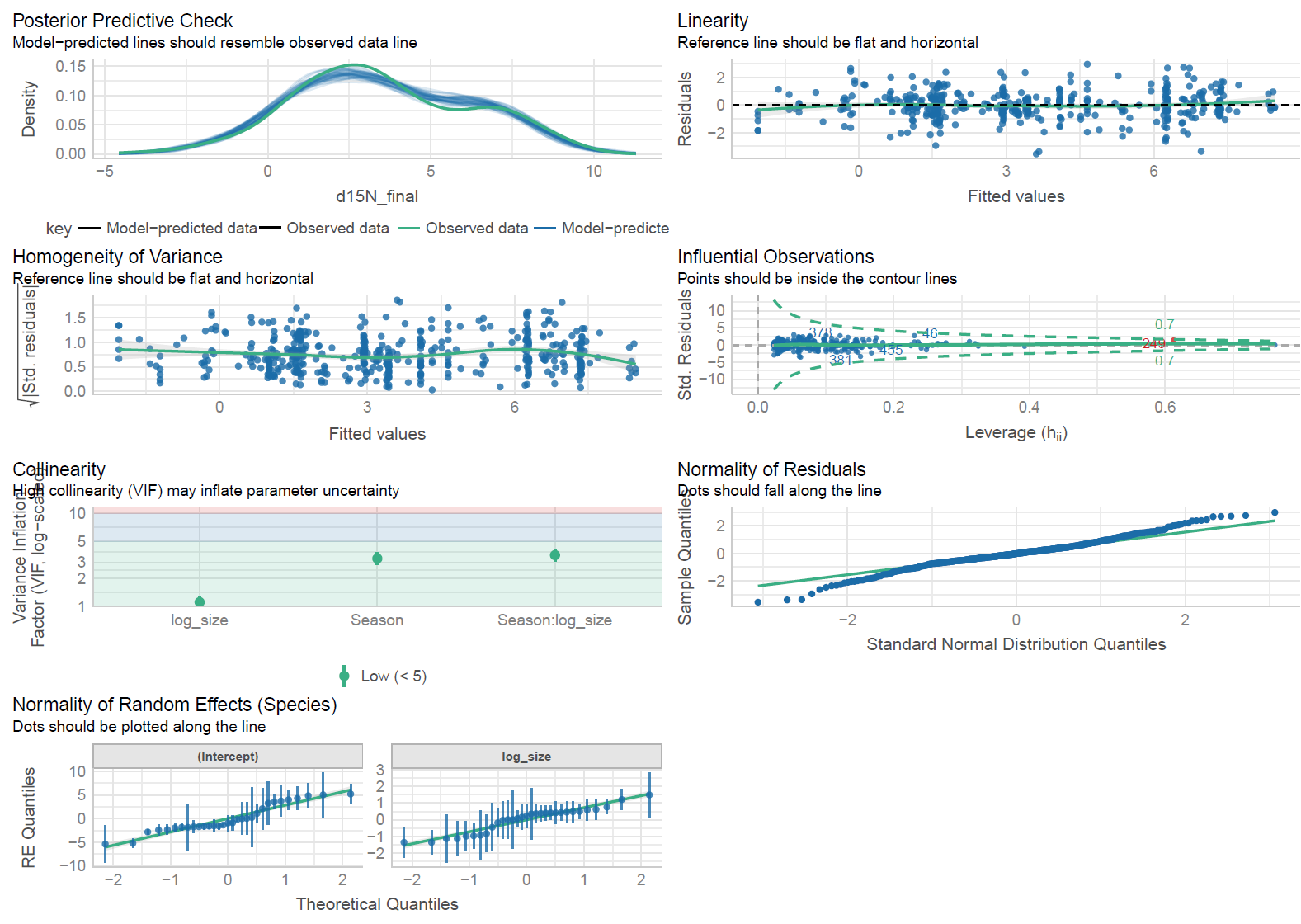


**Figure S11 : : Performance plots for the selected LMEM (model1) on δ^15^N_adjusted_ values.**


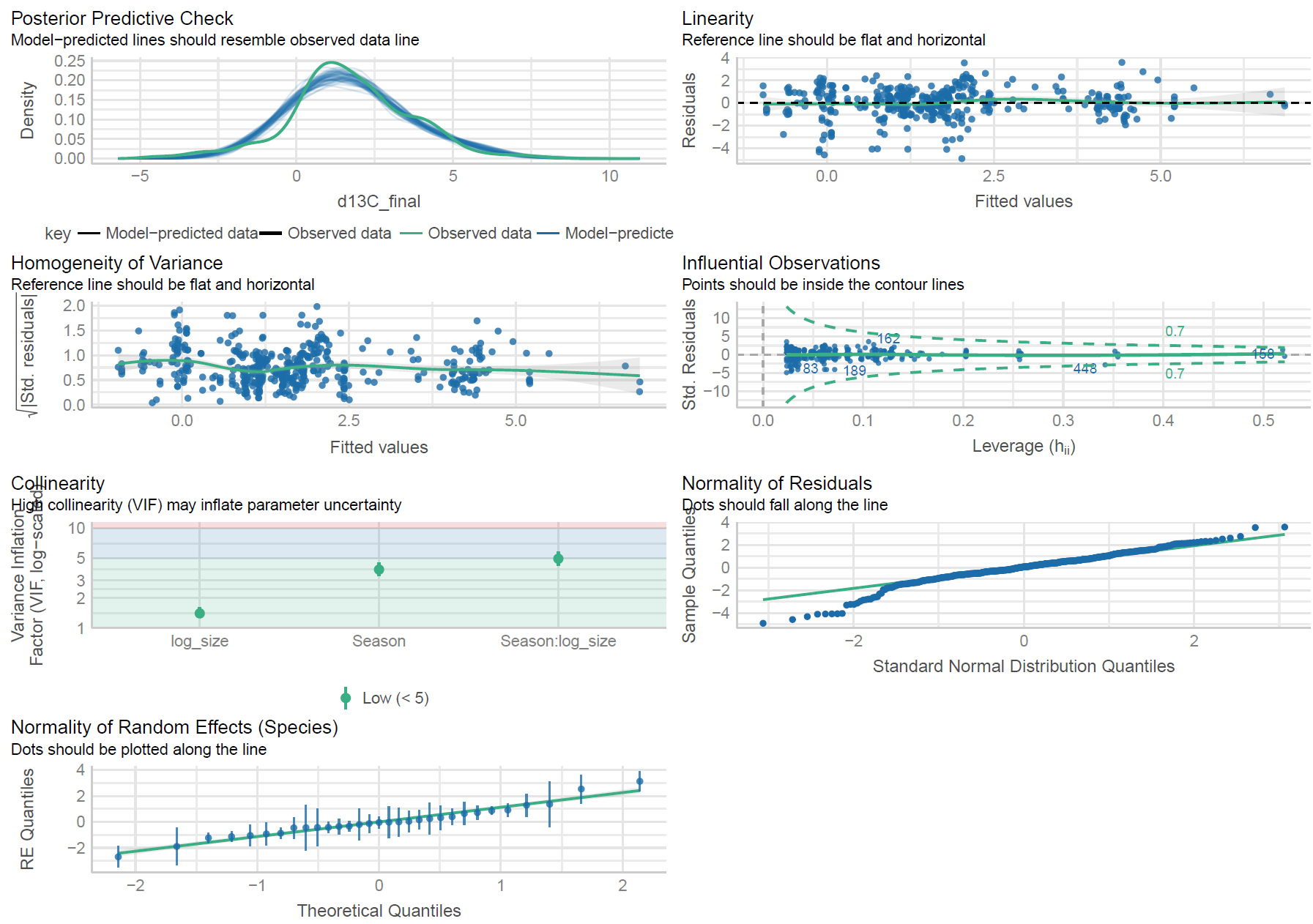


**Figure S12: Performance plots for the selected LMEM (model1) on δ^13^C_adjusted_ values.**


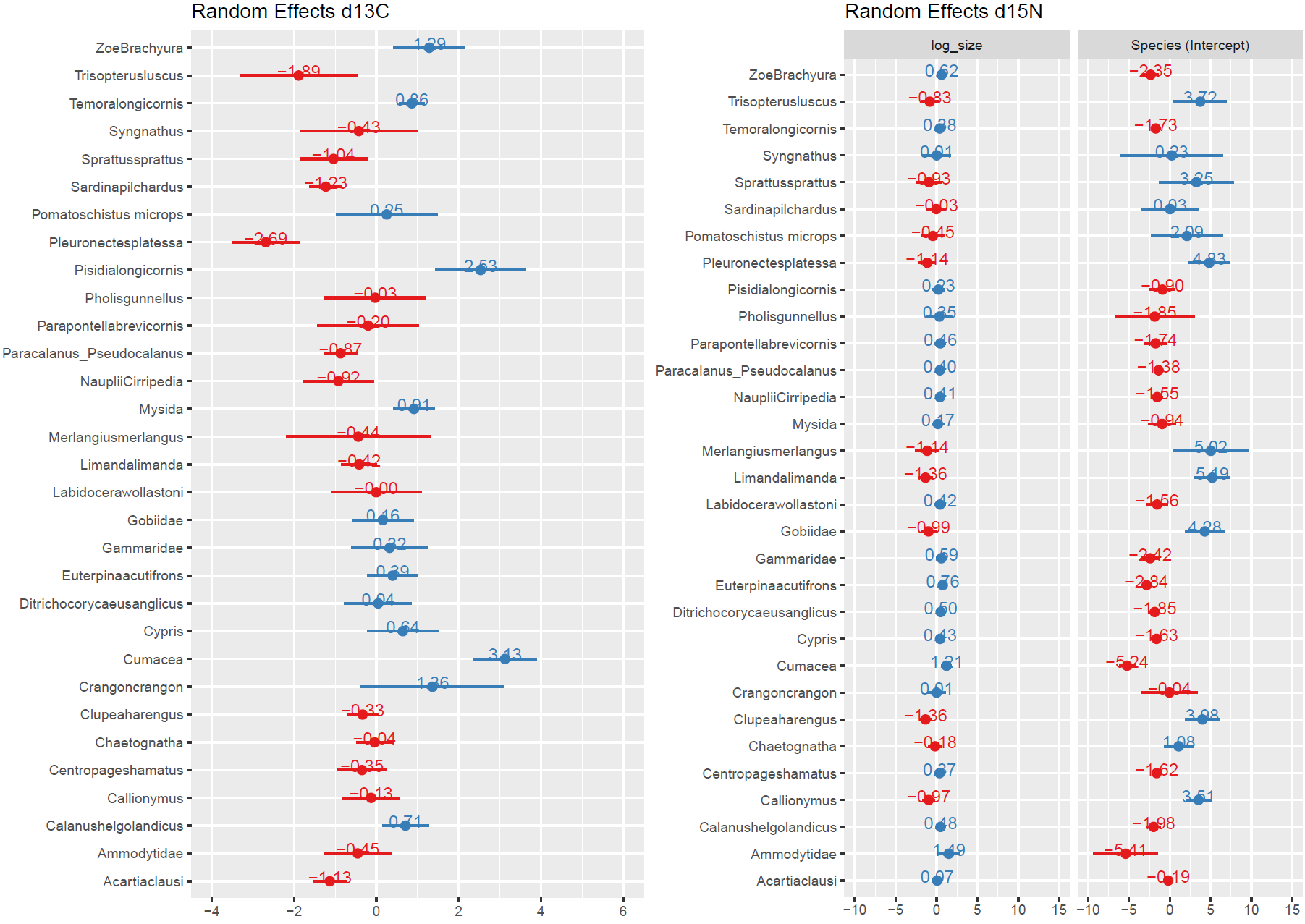


**Figure S13: Random effect structures of the LMEM models.** The values are the difference between the general estimate of the model and the specific level of random effect for a given species/taxa, and size (only for nitrogen). These values capture the variability at each level of the model allowing us to account for species-specific and size-specific effects on the response variable.

# **Supplementary Section S3**

# **« Plankton Topology of the English Channel and Southern Bight »**

**Table S2 : References used to identify potential trophic links (ie., documented interactions, TPlank0) between predator and prey species.**

|  | **Species** | **References used for trophic links** |
| --- | --- | --- |
| **Seston** | POM |  |
| **Copepoda** | *Acartia clausi* | Djeghri, N., Atkinson, A., Fileman, E. S., Harmer, R. A., Widdicombe, C. E., McEvoy, A. J., ... & Mayor, D. J. (2018). High prey-predator size ratios and unselective feeding in copepods: a seasonal comparison of five species with contrasting feeding modes. *Progress in Oceanography*, *165*, 63-74. |
|  |  | Turner, Jefferson T., and Edna Granéli. "Zooplankton feeding ecology: grazing during enclosure studies of phytoplankton blooms from the west coast of Sweden." *Journal of Experimental Marine Biology and Ecology* 157.1 (1992): 19-31. |
|  |  | Wiadnyana, N. N., & Rassoulzadegan, F. (1989). Selective feeding of Acartia clausi and Centropages typicus on microzooplankton. Marine ecology progress series. Oldendorf, 53(1), 37-45. |
|  | *Calanus helgolandicus* | Djeghri, N., Atkinson, A., Fileman, E. S., Harmer, R. A., Widdicombe, C. E., McEvoy, A. J., ... & Mayor, D. J. (2018). High prey-predator size ratios and unselective feeding in copepods: a seasonal comparison of five species with contrasting feeding modes. *Progress in Oceanography*, *165*, 63-74. |
|  |  | Kleppel, G. S. "On the diets of calanoid copepods." *Marine Ecology-Progress Series* 99 (1993): 183-183. |
|  | *Centropages hamatus* | Djeghri, N., Atkinson, A., Fileman, E. S., Harmer, R. A., Widdicombe, C. E., McEvoy, A. J., ... & Mayor, D. J. (2018). High prey-predator size ratios and unselective feeding in copepods: a seasonal comparison of five species with contrasting feeding modes. *Progress in Oceanography*, *165*, 63-74. |
|  |  | Conley, Walter J., and Jefferson T. Turner. "Omnivory by the coastal marine copepods Centropages hamatus and Labidocera aestiva." *Marine ecology progress series. Oldendorf* 21.1 (1985): 113-120. |
|  |  | Turner, Jefferson T., and Edna Granéli. "Zooplankton feeding ecology: grazing during enclosure studies of phytoplankton blooms from the west coast of Sweden." *Journal of Experimental Marine Biology and Ecology* 157.1 (1992): 19-31. |
|  | *Ditrichocorycaeus anglicus* | Landry, M. R., & Fagerness, V. L. (1988). Behavioral and morphological influences on predatory interactions among marine copepods. *Bulletin of Marine Science*, *43*(3), 509-529. |
|  | *Euterpina acutifrons* | Sautour, B., & Castel, J. (1993). Feeding behaviour of the coastal copepod Euterpina acutifrons on small particles. *Cah. Biol. Mar*, *34*(2), 239-251. |
|  |  | Dıaz, E., Cotano, U., & Villate, F. (2003). Reproductive response of Euterpina acutifrons in two estuaries of the Basque Country (Bay of Biscay) with contrasting nutritional environment. *Journal of experimental marine biology and ecology*, *292*(2), 213-230. |
|  | *Labidocera wollastoni* | Lindley, J. A., & Hunt, H. G. (1989). The distributions of Labidocera wollastoni and Centropages hamatus in the North Atlantic Ocean and the North Sea in relation to the role of resting eggs in the sediment. *Reproduction, genetics and distributions of marine organisms*, *104*(2), 407-413. |
|  |  | Ohtsuka, S., & Onbé, T. (1991). Relationship between mouthpart structures and in situ feeding habits of species of the family Pontellidae (Copepoda: Calanoida). *Marine Biology*, *111*(2), 213-225. |
|  | Paracalanus sp/ Pseudocalanus sp | Djeghri, N., Atkinson, A., Fileman, E. S., Harmer, R. A., Widdicombe, C. E., McEvoy, A. J., ... & Mayor, D. J. (2018). High prey-predator size ratios and unselective feeding in copepods: a seasonal comparison of five species with contrasting feeding modes. *Progress in Oceanography*, *165*, 63-74. |
|  | *Parapontella brevicornis* | Benedetti, F., Gasparini, S., & Ayata, S. D. (2016). Identifying copepod functional groups from species functional traits. *Journal of Plankton Research*, *38*(1), 159-166. |
|  | *Temora longicornis* | Kleppel, G. S. "On the diets of calanoid copepods." *Marine Ecology-Progress Series* 99 (1993): 183-183. |
|  |  | Harris, R. P., & Paffenhöfer, G. A. (1976). Feeding, growth and reproduction of the marine planktonic copepod Temora longicornis Müller. *Journal of the Marine Biological Association of the United Kingdom*, *56*(3), 675-690. |
|  |  | Dam, H. G., & Lopes, R. M. (2003). Omnivory in the calanoid copepod Temora longicornis: feeding, egg production and egg hatching rates. *Journal of experimental marine biology and ecology*, *292*(2), 119-137. |
|  |  | Evans, F. (1981). An investigation into the relationship of sea temperature and food supply to the size of the planktonic copepod Temora longicornis Müller in the North Sea. *Estuarine, Coastal and Shelf Science*, *13*(2), 145-158. |
|  |  | Gentsch, E., Kreibich, T., Hagen, W., & Niehoff, B. (2009). Dietary shifts in the copepod Temora longicornis during spring: evidence from stable isotope signatures, fatty acid biomarkers and feeding experiments. *Journal of Plankton Research*, *31*(1), 45-60. |
| **Malacostraca** | *Crangon crangon* | Criales, M. M., & Anger, K. (1986). Experimental studies on the larval development of the shrimps Crangon crangon and C. allmanni. *Helgoländer Meeresuntersuchungen*, *40*(3), 241-265. |
|  |  | Paschke, K. A., Gebauer, P., Buchholz, F., & Anger, K. (2004). Seasonal variation in starvation resistance of early larval North Sea shrimp Crangon crangon (Decapoda: Crangonidae). *Marine Ecology Progress Series*, *279*, 183-191. |
|  | *Pisidia longicornis* | Harms, J., & Seeger, B. (1989). Larval development and survival in seven decapod species (Crustacea) in relation to laboratory diet. *Journal of experimental marine biology and ecology*, *133*(1-2), 129-139. |
|  |  | Anger, K. (2001). *The biology of decapod crustacean larvae* (Vol. 14, pp. 1-420). Lisse: AA Balkema Publishers. |
|  | Cumacea | Watling, L.; Gerken, S. (2020). World Cumacea Database. Accessed at http://www.marinespecies.org/cumacea on 2020-08-07. doi:10.14284/354 |
|  | Gammaridae | Glazier, D. S. (2014). Amphipoda. Encyclopedia of Inland Waters, 2009, Pages 89-115 |
|  |  | Mancinelli, G. (2012). On the trophic ecology of Gammaridea (Crustacea: Amphipoda) in coastal waters: a European-scale analysis of stable isotopes data. *Estuarine, Coastal and Shelf Science*, *114*, 130-139. |
|  | Mysida | Mauchline, J. (1980). Blaxter, J. H. S.; Russell, F. S.; Yonge, M. (eds.). *The Biology of Mysids and Euphausiids*. Advances in Marine Biology: Volume 18. Academic Press. ISBN 978-0-08-057941-2. |
|  |  | Viherluoto, M. (2001). Food selection and feeding behaviour of Baltic Sea mysid shrimps. |
|  | Zoe Brachyura | Harms, J., & Seeger, B. (1989). Larval development and survival in seven decapod species (Crustacea) in relation to laboratory diet. *Journal of experimental marine biology and ecology*, *133*(1-2), 129-139. |
|  |  | Rhyne, A. L., Penha-Lopes, G., & Lin, J. (2005). Growth, development, and survival of larval Mithraculus sculptus (Lamark) and Mithraculus forceps (A. Milne Edwards)(Decapoda: Brachyura: Majidae): economically important marine ornamental crabs. *Aquaculture*, *245*(1-4), 183-191. |
|  |  | Anger, K. (2001). *The biology of decapod crustacean larvae* (Vol. 14, pp. 1-420). Lisse: AA Balkema Publishers. |
| **Cirripedia** | Nauplii Cirripedia | Anger, K. (2001). *The biology of decapod crustacean larvae* (Vol. 14, pp. 1-420). Lisse: AA Balkema Publishers. |
|  | Cypris | Anger, K. (2001). *The biology of decapod crustacean larvae* (Vol. 14, pp. 1-420). Lisse: AA Balkema Publishers. |
|  |  | HØEG, J. T., & MØLLER, O. S. (2006). When similar beginnings lead to different ends: constraints and diversity in cirripede larval development. *Invertebrate Reproduction & Development*, *49*(3), 125-142. |
| **Chaetognatha** | Chaetognatha (*Sagitta elegans; Parasagitta setosa*) | Frid, C. L. J., Newton, L. C., & Williams, J. A. (1994). The feeding rates ofPleurobrachia (ctenophora) andSagitta (chaetognatha), with notes on the potential seasonal role of planktonic predators in the dynamics of north sea zooplankton communities. *Netherland Journal of Aquatic Ecology*, *28*(2), 181-191. |
|  |  | Alvarez-Cadena, J. N. (1993). Feeding of the chaetognath Sagitta elegans Verrill. *Estuarine, Coastal and Shelf Science*, *36*(2), 195-206. |
|  |  | Clark, R. A., Frid, C. L., & Nicholas, K. R. (2003). Long-term, predation-based control of a central-west North Sea zooplankton community. *ICES Journal of Marine Science*, *60*(2), 187-197. |
| **Actinopterygii** | Ammodytidae (*Ammodytes tobianus*) | Lebour, Marie V. "The food of post-larval fish." *Journal of the Marine Biological Association of the United Kingdom* 12.1 (1919): 22-47. |
|  | *Clupea herringus* | Denis, J., Vallet, C., Courcot, L., Lefebvre, V., Caboche, J., Antajan, E., ... & Loots, C. (2016). Feeding strategy of Downs herring larvae (Clupea herringus L.) in the English Channel and North Sea. *Journal of Sea Research*, *115*, 33-46. |
|  |  | Lebour, Marie V. "The food of post-larval fish." *Journal of the Marine Biological Association of the United Kingdom* 12.1 (1919): 22-47. |
|  | Callionymus (C. lyra) | Lebour, Marie V. "The food of post-larval fish." *Journal of the Marine Biological Association of the United Kingdom* 12.1 (1919): 22-47. |
|  | Gobiidae (*Pomatoschistus minutus, Pomatoschistus lozanoi, Gobiusculus flavescens, Gobius paganellus* ) | Hostens, Kristian, and Jan Mees. "The mysid‐feeding guild of demersal fishes in the brackish zone of the Westerschelde estuary." *Journal of Fish Biology* 55.4 (1999): 704-719. |
|  |  | Lebour, Marie V. "The food of post-larval fish." *Journal of the Marine Biological Association of the United Kingdom* 12.1 (1919): 22-47. |
|  | *Limanda limanda* | Last, J. M. (1978). The food of four species of pleuronectiform larvae in the eastern English Channel and southern North Sea. *Marine Biology*, *45*(4), 359-368. |
|  | *Merlangius merlangus* | Last, J. M. (1978). The food of three species of gadoid larvae in the eastern English Channel and southern North Sea. *Marine Biology*, *48*(4), 377-386. |
|  |  | Economou, A. N. (1991). Food and feeding ecology of five gadoid larvae in the northern North Sea. *ICES Journal of Marine Science*, *47*(3), 339-351. |
|  |  | Lebour, Marie V. "The food of post-larval fish." *Journal of the Marine Biological Association of the United Kingdom* 12.1 (1919): 22-47. |
|  | *Sprattus sprattus* | Voss, R., Dickmann, M., & Schmidt, J. O. (2009). Feeding ecology of sprat (Sprattus sprattus L.) and sardine (Sardina pilchardus W.) larvae in the German Bight, North Sea. *Oceanologia*, *51*(1), 117-138. |
|  |  | Lebour, Marie V. "The food of post-larval fish." *Journal of the Marine Biological Association of the United Kingdom* 12.1 (1919): 22-47. |
|  | *Trisopterus luscus* | Last, J. M. (1978). The food of three species of gadoid larvae in the eastern English Channel and southern North Sea. *Marine Biology*, *48*(4), 377-386. |
|  |  | Hostens, Kristian, and Jan Mees. "The mysid‐feeding guild of demersal fishes in the brackish zone of the Westerschelde estuary." *Journal of Fish Biology* 55.4 (1999): 704-719. |
|  | Syngnathus (*S. rostellatus, S. acus*) | <https://www.fishbase.se/TrophicEco/FoodItemsList.php?vstockcode=1377&genus=Syngnathus&species=rostellatus> |
|  |  | <https://www.fishbase.se/trophiceco/DietCompoSummary.php?dietcode=4315&genusname=Syngnathus&speciesname=acus> |
|  |  | Lebour, Marie V. "The food of post-larval fish." *Journal of the Marine Biological Association of the United Kingdom* 12.1 (1919): 22-47. |
|  | *Pomatoschistus microps* | Jackson, A. C., and S. D. Rundle. "Diet–shifts by an estuarine goby (Pomatoschistus microps) in the face of variable prey availability." *Journal of Experimental Marine Biology and Ecology* 361.1 (2008): 1-7. |
|  |  | Mendonca, V., Raffaelli, D., Boyle, P., & Emes, C. (2007). The ecological role of overwintering fish in the food web of the Culbin Sands lagoon ecosystem, NE Scotland: Identifying major trophic links and testing effects of the fish Pomatoschistus microps (Pallas) on benthic invertebrates. *Scientia Marina*, *71*(4), 649-660. |
|  |  | Baeta, Alexandra, et al. "Use of stable isotope ratios of fish larvae as indicators to assess diets and patterns of anthropogenic nitrogen pollution in estuarine ecosystems." *Ecological Indicators* 83 (2017): 112-121. |
|  |  | Hostens, Kristian, and Jan Mees. "The mysid‐feeding guild of demersal fishes in the brackish zone of the Westerschelde estuary." *Journal of Fish Biology* 55.4 (1999): 704-719. |
|  | *Sardina pilchardus* | Voss, R., Dickmann, M., & Schmidt, J. O. (2009). Feeding ecology of sprat (Sprattus sprattus L.) and sardine (Sardina pilchardus W.) larvae in the German Bight, North Sea. *Oceanologia*, *51*(1), 117-138. |
|  |  | Morote, E., Olivar, M. P., Villate, F., & Uriarte, I. (2010). A comparison of anchovy (Engraulis encrasicolus) and sardine (Sardina pilchardus) larvae feeding in the Northwest Mediterranean: influence of prey availability and ontogeny. *ICES Journal of Marine Science*, *67*(5), 897-908. |
|  | *Pleuronectes platessa* | Last, J. M. (1978). The food of four species of pleuronectiform larvae in the eastern English Channel and southern North Sea. *Marine Biology*, *45*(4), 359-368. |
|  |  | Mendonca, V., Raffaelli, D., Boyle, P., & Emes, C. (2007). The ecological role of overwintering fish in the food web of the Culbin Sands lagoon ecosystem, NE Scotland: Identifying major trophic links and testing effects of the fish Pomatoschistus microps (Pallas) on benthic invertebrates. *Scientia Marina*, *71*(4), 649-660. |
|  |  | Hostens, Kristian, and Jan Mees. "The mysid‐feeding guild of demersal fishes in the brackish zone of the Westerschelde estuary." *Journal of Fish Biology* 55.4 (1999): 704-719. |
|  | *Pholis gunnellus* | Cheetham, C., & Fives, J. M. (1990, January). The biology and parasites of the butterfish Pholis gunnellus (Linnaeus, 1758) in the Galway Bay area. In *Proceedings of the Royal Irish Academy. Section B: Biological, Geological, and Chemical Science* (pp. 127-149). Royal Irish Academy. |

**Table S3 : Predator-prey ratios used to infer possible trophic links for zooplankton species when information was unavailable (TPlank1).** Based on the equation proposed by Djeghri, N. et al. (2018) High prey-predator size ratios and unselective feeding in copepods: A seasonal comparison of five species with contrasting feeding modes. Prog. Oceanogr. 165, 63–74. Note : for carnivorous decapod larvae, the upper threshold prey size approximates or may even exceed the body size of the predator. Values in parenthesis for each species indicate the calculated maximum value to infer predation.

| **Zooplankton** | **Max total lenght** | **Min total length** | ***Acartia clausi* (0.45)** | ***Calanus helgolandicus* (0.14)** | **Para_Pseudo calanus (0.18)** | **Chaetognatha (0.4)** | ***Ditrichocorycaeus anglicus* (1.25)** | **Zoe Brachyura (1)** |
| --- | --- | --- | --- | --- | --- | --- | --- | --- |
| *Acartia clausi* | 1.44 | 0.9 |  | 0.30 | 0.67 | 0.09 | 0.88 | 0.30 |
| *Calanus helgolandicus* | 3 | 2.12 | 1.48 |  | 1.59 | 0.22 | 2.08 | 0.70 |
| *Centropages hamatus* | 1.7 | 0.99 | 0.69 | 0.33 | 0.74 | 0.10 | 0.97 | 0.33 |
| Chaetognatha | 9.72 | 4.2 | 2.92 | 1.40 | 3.13 |  | 4.12 | 1.39 |
| *Crangon crangon* | 5.75 | 5.75 | 3.99 | 1.92 | 4.29 | 0.59 | 5.64 | 1.90 |
| Cumacea | 3.94 | 1.43 | 1.00 | 0.48 | 1.07 | 0.15 | 1.41 | 0.48 |
| Cypris | 0.86 | 0.78 | 0.54 | 0.26 | 0.58 | 0.08 | 0.76 | 0.26 |
| *Ditrichocorycaeus anglicus* | 1.02 | 0.77 | 0.54 | 0.26 | 0.58 | 0.08 |  | 0.26 |
| *Euterpina acutifrons* | 0.66 | 0.55 | 0.38 | 0.18 | 0.41 | 0.06 | 0.54 | 0.18 |
| Gammaridae | 3.06 | 1.20 | 0.83 | 0.40 | 0.90 | 0.12 | 1.18 | 0.40 |
| *Labidocera wollastoni* | 2.35 | 2.08 | 1.45 | 0.69 | 1.55 | 0.21 | 2.04 | 0.69 |
| Mysida | 10.9 | 2.5 | 1.74 | 0.83 | 1.87 | 0.26 | 2.45 | 0.83 |
| Nauplii Cirripedia | 0.47 | 0.42 | 0.29 | 0.14 | 0.31 | 0.04 | 0.41 | 0.14 |
| *Paracalanus_Pseudocalanus* | 1.34 | 0.69 | 0.47 | 0.23 |  | 0.07 | 0.67 | 0.23 |
| *Parapontella brevicornis* | 1.84 | 1.70 | 1.18 | 0.57 | 1.27 | 0.17 | 1.67 | 0.56 |
| *Pisidia longicornis* | 3.34 | 2.62 | 1.82 | 0.87 | 1.96 | 0.27 | 2.57 | 0.87 |
| *Temora longicornis* | 1.88 | 0.56 | 0.39 | 0.19 | 0.42 | 0.06 | 0.55 | 0.19 |
| Zoe Brachyura | 3.02 | 1.53 | 1.06 | 0.51 | 1.14 | 0.16 | 1.50 |  |

**Table S4 : Summary of seasonal predator-prey interactions for the MixSIAR models.** Species are ordered alphabetically based on prey. Species that were indistinguishable based on their stable isotopes values and had similar sizes were combined (e.g., 'Chel_Plo' represents the combined prey group of C. helgolandicus and P. longicornis). Prey are abbreviated using the first letter of the genus and the first three letters of the species (e.g., 'Aclau' refers to Acartia clausi)

| **Predator** | **Season** | **Prey (p)** |
| --- | --- | --- |
| chaetognathe | autumn | p.Aclau |
| sardine | autumn | p.Aclau |
| callionimes | spring | p.Aclau |
| herring | spring | p.Aclau |
| limande | spring | p.Aclau |
| mysida | spring | p.Aclau |
| sprat | spring | p.Aclau |
| chaetognathe | winter | p.Aclau |
| herring | winter | p.Aclau |
| mysida | winter | p.Aclau |
| plaice | winter | p.Aclau |
| sardine | winter | p.Aclau |
| chaetognathe | autumn | p.Chama |
| sardine | autumn | p.Chama |
| callionimes | spring | p.Chama |
| herring | spring | p.Chama |
| limande | spring | p.Chama |
| mysida | spring | p.Chama |
| sprat | spring | p.Chama |
| chaetognathe | autumn | p.Che_Plo |
| sardine | autumn | p.Chel_Plo |
| callionimes | spring | p.Chelg |
| herring | spring | p.Chelg |
| limande | spring | p.Chelg |
| mysida | spring | p.Chelg |
| sprat | spring | p.Chelg |
| chaetognathe | winter | p.Chelg |
| herring | winter | p.Chelg |
| mysida | winter | p.Chelg |
| plaice | winter | p.Chelg |
| sardine | winter | p.Chelg |
| chaetognathe | autumn | p.Cuma |
| sardine | autumn | p.Cuma |
| chaetognathe | winter | p.Cuma |
| herring | winter | p.Cuma |
| sardine | winter | p.Cuma |
| herring | spring | p.Cyprr |
| mysida | spring | p.Cyprr |
| sprat | spring | p.Cyprr |
| chaetognathe | autumn | p.Dan_PaPs |
| sardine | autumn | p.Dan_PaPs |
| chaetognathe | autumn | p.Eacut |
| sardine | autumn | p.Eacut |
| callionimes | spring | p.Eacut |
| herring | spring | p.Eacut |
| limande | spring | p.Eacut |
| mysida | spring | p.Eacut |
| sprat | spring | p.Eacut |
| chaetognathe | autumn | p.Lwoll |
| sardine | autumn | p.Lwoll |
| chaetognathe | autumn | p.Malacostraca |
| sardine | autumn | p.Malacostraca |
| herring | spring | p.Ncirr |
| limande | spring | p.Ncirr |
| mysida | spring | p.Ncirr |
| sprat | spring | p.Ncirr |
| callionimes | spring | p.PaPse |
| herring | spring | p.PaPse |
| limande | spring | p.PaPse |
| mysida | spring | p.PaPse |
| sprat | spring | p.PaPse |
| chaetognathe | winter | p.PaPse |
| herring | winter | p.PaPse |
| mysida | winter | p.PaPse |
| plaice | winter | p.PaPse |
| sardine | winter | p.PaPse |
| callionimes | spring | p.Pbrev |
| herring | spring | p.Pbrev |
| limande | spring | p.Pbrev |
| mysida | spring | p.Pbrev |
| sprat | spring | p.Pbrev |
| chaetognathe | autumn | p.POM |
| herring | spring | p.POM |
| limande | spring | p.POM |
| mysida | spring | p.POM |
| chaetognathe | winter | p.POM |
| herring | winter | p.POM |
| mysida | winter | p.POM |
| plaice | winter | p.POM |
| chaetognathe | autumn | p.Tlong |
| sardine | autumn | p.Tlong |
| callionimes | spring | p.Tlong |
| herring | spring | p.Tlong |
| limande | spring | p.Tlong |
| mysida | spring | p.Tlong |
| sprat | spring | p.Tlong |
| chaetognathe | winter | p.Tlong |
| herring | winter | p.Tlong |
| mysida | winter | p.Tlong |
| plaice | winter | p.Tlong |
| sardine | winter | p.Tlong |

**Tables S5 to Table S10 : Topology (Trophic interactions)**. Seasonal topologies are constructed based on documented iteractions (TPlank0, from either peer-reviewed publications, gray literature or institutional reports, as reported on Table S4). These were completed by inference, on the basis of knowledge on similar species from comparable regions and maximum prey/predator ratios (length of the largest prey divided by the length of the predator (potential interactions, TPlank1) as reported on Table S5.

**Table S5 : Winter TPlank0**

| **POM** | **Aclau** | **Chelg** | **Chama** | **Eacut** | **PaPse** | **Tlong** | **Ccran** | **Cuma** | **Gamm** | **Mysi** | **Chaet** | **Ammo** | **Chare** | **Spilc** | **Pplat** |
| --- | --- | --- | --- | --- | --- | --- | --- | --- | --- | --- | --- | --- | --- | --- | --- |
| 0 | 1 | 1 | 1 | 1 | 1 | 1 | 1 | 1 | 1 | 1 | 1 | 1 | 1 | 0 | 1 |
| 0 | 0 | 0 | 0 | 0 | 0 | 0 | 0 | 0 | 0 | 0 | 1 | 0 | 0 | 1 | 0 |
| 0 | 0 | 0 | 0 | 0 | 0 | 0 | 0 | 0 | 0 | 0 | 1 | 0 | 0 | 0 | 0 |
| 0 | 0 | 0 | 0 | 0 | 0 | 0 | 0 | 0 | 0 | 0 | 1 | 0 | 0 | 1 | 0 |
| 0 | 0 | 0 | 0 | 0 | 0 | 0 | 0 | 0 | 0 | 0 | 0 | 0 | 1 | 0 | 0 |
| 0 | 0 | 0 | 0 | 0 | 0 | 0 | 0 | 0 | 0 | 0 | 1 | 1 | 1 | 1 | 1 |
| 0 | 0 | 0 | 0 | 0 | 0 | 0 | 0 | 0 | 0 | 1 | 1 | 0 | 1 | 1 | 0 |
| 0 | 0 | 0 | 0 | 0 | 0 | 0 | 0 | 0 | 0 | 0 | 0 | 0 | 0 | 0 | 0 |
| 0 | 0 | 0 | 0 | 0 | 0 | 0 | 0 | 0 | 0 | 0 | 0 | 0 | 0 | 0 | 0 |
| 0 | 0 | 0 | 0 | 0 | 0 | 0 | 0 | 0 | 0 | 0 | 0 | 0 | 0 | 0 | 0 |
| 0 | 0 | 0 | 0 | 0 | 0 | 0 | 0 | 0 | 0 | 0 | 0 | 0 | 0 | 0 | 0 |
| 0 | 0 | 0 | 0 | 0 | 0 | 0 | 0 | 0 | 0 | 0 | 0 | 0 | 0 | 0 | 0 |
| 0 | 0 | 0 | 0 | 0 | 0 | 0 | 0 | 0 | 0 | 0 | 0 | 0 | 0 | 0 | 0 |
| 0 | 0 | 0 | 0 | 0 | 0 | 0 | 0 | 0 | 0 | 0 | 0 | 0 | 0 | 0 | 0 |
| 0 | 0 | 0 | 0 | 0 | 0 | 0 | 0 | 0 | 0 | 0 | 0 | 0 | 0 | 0 | 0 |
| 0 | 0 | 0 | 0 | 0 | 0 | 0 | 0 | 0 | 0 | 0 | 0 | 0 | 0 | 0 | 0 |

**Table S6 : Winter TPlank1**

| **POM** | **Aclau** | **Chelg** | **Chama** | **Eacut** | **PaPse** | **Tlong** | **Ccran** | **Cuma** | **Gamm** | **Mysi** | **Chaet** | **Ammo** | **Chare** | **Spilc** | **Pplat** |
| --- | --- | --- | --- | --- | --- | --- | --- | --- | --- | --- | --- | --- | --- | --- | --- |
| 0 | 1 | 1 | 1 | 1 | 1 | 1 | 1 | 1 | 1 | 1 | 1 | 1 | 1 | 0 | 1 |
| 0 | 0 | 0 | 0 | 0 | 0 | 0 | 0 | 0 | 0 | 1 | 1 | 0 | 1 | 1 | 1 |
| 0 | 0 | 0 | 0 | 0 | 0 | 0 | 0 | 0 | 0 | 1 | 1 | 0 | 1 | 1 | 1 |
| 0 | 0 | 0 | 0 | 0 | 0 | 0 | 0 | 0 | 0 | 1 | 1 | 0 | 1 | 1 | 1 |
| 0 | 1 | 0 | 0 | 0 | 0 | 0 | 0 | 0 | 0 | 1 | 1 | 0 | 1 | 1 | 1 |
| 0 | 0 | 0 | 0 | 0 | 0 | 0 | 0 | 0 | 0 | 1 | 1 | 1 | 1 | 1 | 1 |
| 0 | 1 | 0 | 0 | 0 | 0 | 0 | 0 | 0 | 0 | 1 | 1 | 0 | 1 | 1 | 1 |
| 0 | 0 | 0 | 0 | 0 | 0 | 0 | 0 | 0 | 0 | 0 | 0 | 0 | 1 | 1 | 0 |
| 0 | 0 | 0 | 0 | 0 | 0 | 0 | 0 | 0 | 0 | 0 | 1 | 0 | 1 | 1 | 0 |
| 0 | 0 | 0 | 0 | 0 | 0 | 0 | 0 | 0 | 0 | 0 | 1 | 0 | 1 | 1 | 0 |
| 0 | 0 | 0 | 0 | 0 | 0 | 0 | 0 | 0 | 0 | 0 | 0 | 0 | 1 | 1 | 0 |
| 0 | 0 | 0 | 0 | 0 | 0 | 0 | 0 | 0 | 0 | 0 | 0 | 0 | 0 | 0 | 0 |
| 0 | 0 | 0 | 0 | 0 | 0 | 0 | 0 | 0 | 0 | 0 | 0 | 0 | 0 | 0 | 0 |
| 0 | 0 | 0 | 0 | 0 | 0 | 0 | 0 | 0 | 0 | 0 | 1 | 0 | 0 | 0 | 0 |
| 0 | 0 | 0 | 0 | 0 | 0 | 0 | 0 | 0 | 0 | 0 | 0 | 0 | 0 | 0 | 0 |
| 0 | 0 | 0 | 0 | 0 | 0 | 0 | 0 | 0 | 0 | 0 | 0 | 0 | 0 | 0 | 0 |

**Table S7 : Spring Tplank0**

| **POM** | **Aclau** | **Chelg** | **Chama** | **Eacut** | **PaPse** | **Pbrev** | **Tlong** | **Cuma** | **Mysi** | **Brach** | **Ncirr** | **Cyprr** | **Ammo** | **Chare** | **Calli** | **Gobbi** | **Llima** | **Mmerl** | **Sspra** | **Tlusc** | **Spilc** | **Pgunn** |
| --- | --- | --- | --- | --- | --- | --- | --- | --- | --- | --- | --- | --- | --- | --- | --- | --- | --- | --- | --- | --- | --- | --- |
| 0 | 1 | 1 | 1 | 1 | 1 | 1 | 1 | 1 | 1 | 1 | 1 | 0 | 1 | 1 | 0 | 0 | 1 | 1 | 1 | 1 | 0 | 0 |
| 0 | 0 | 0 | 0 | 0 | 0 | 0 | 0 | 0 | 0 | 0 | 0 | 0 | 0 | 0 | 1 | 1 | 1 | 1 | 1 | 1 | 1 | 0 |
| 0 | 0 | 0 | 0 | 0 | 0 | 0 | 0 | 0 | 0 | 0 | 0 | 0 | 0 | 0 | 0 | 1 | 0 | 0 | 0 | 0 | 0 | 0 |
| 0 | 0 | 0 | 0 | 0 | 0 | 0 | 0 | 0 | 0 | 0 | 0 | 0 | 0 | 0 | 0 | 1 | 0 | 0 | 1 | 0 | 1 | 0 |
| 0 | 0 | 0 | 0 | 0 | 0 | 0 | 0 | 0 | 0 | 0 | 0 | 0 | 0 | 1 | 0 | 1 | 0 | 0 | 0 | 0 | 0 | 0 |
| 0 | 0 | 0 | 0 | 0 | 0 | 0 | 0 | 0 | 0 | 0 | 0 | 0 | 1 | 1 | 1 | 1 | 1 | 1 | 1 | 1 | 1 | 0 |
| 0 | 0 | 0 | 0 | 0 | 0 | 0 | 0 | 0 | 0 | 0 | 0 | 0 | 0 | 0 | 0 | 1 | 0 | 0 | 0 | 0 | 0 | 0 |
| 0 | 0 | 0 | 0 | 0 | 0 | 0 | 0 | 0 | 1 | 0 | 0 | 0 | 0 | 1 | 1 | 1 | 1 | 1 | 1 | 0 | 1 | 0 |
| 0 | 0 | 0 | 0 | 0 | 0 | 0 | 0 | 0 | 0 | 0 | 0 | 0 | 0 | 0 | 0 | 1 | 0 | 0 | 0 | 0 | 0 | 0 |
| 0 | 0 | 0 | 0 | 0 | 0 | 0 | 0 | 0 | 0 | 0 | 0 | 0 | 0 | 0 | 0 | 1 | 0 | 0 | 0 | 1 | 0 | 0 |
| 0 | 0 | 0 | 0 | 0 | 0 | 0 | 0 | 0 | 0 | 0 | 0 | 0 | 0 | 0 | 0 | 1 | 0 | 0 | 0 | 0 | 0 | 0 |
| 0 | 0 | 0 | 0 | 0 | 0 | 0 | 0 | 0 | 0 | 0 | 0 | 0 | 1 | 1 | 0 | 1 | 1 | 1 | 1 | 0 | 0 | 0 |
| 0 | 0 | 0 | 0 | 0 | 0 | 0 | 0 | 0 | 0 | 0 | 0 | 0 | 1 | 0 | 0 | 1 | 0 | 1 | 1 | 0 | 0 | 0 |
| 0 | 0 | 0 | 0 | 0 | 0 | 0 | 0 | 0 | 0 | 0 | 0 | 0 | 0 | 0 | 0 | 0 | 0 | 0 | 0 | 0 | 0 | 0 |
| 0 | 0 | 0 | 0 | 0 | 0 | 0 | 0 | 0 | 0 | 0 | 0 | 0 | 0 | 0 | 0 | 0 | 0 | 0 | 0 | 0 | 0 | 0 |
| 0 | 0 | 0 | 0 | 0 | 0 | 0 | 0 | 0 | 0 | 0 | 0 | 0 | 0 | 0 | 0 | 0 | 0 | 0 | 0 | 0 | 0 | 0 |
| 0 | 0 | 0 | 0 | 0 | 0 | 0 | 0 | 0 | 0 | 0 | 0 | 0 | 0 | 0 | 0 | 0 | 0 | 0 | 0 | 0 | 0 | 0 |
| 0 | 0 | 0 | 0 | 0 | 0 | 0 | 0 | 0 | 0 | 0 | 0 | 0 | 0 | 0 | 0 | 0 | 0 | 0 | 0 | 0 | 0 | 0 |
| 0 | 0 | 0 | 0 | 0 | 0 | 0 | 0 | 0 | 0 | 0 | 0 | 0 | 0 | 0 | 0 | 0 | 0 | 0 | 0 | 0 | 0 | 0 |
| 0 | 0 | 0 | 0 | 0 | 0 | 0 | 0 | 0 | 0 | 0 | 0 | 0 | 0 | 0 | 0 | 0 | 0 | 0 | 0 | 0 | 0 | 0 |
| 0 | 0 | 0 | 0 | 0 | 0 | 0 | 0 | 0 | 0 | 0 | 0 | 0 | 0 | 0 | 0 | 0 | 0 | 0 | 0 | 0 | 0 | 0 |
| 0 | 0 | 0 | 0 | 0 | 0 | 0 | 0 | 0 | 0 | 0 | 0 | 0 | 0 | 0 | 0 | 0 | 0 | 0 | 0 | 0 | 0 | 0 |
| 0 | 0 | 0 | 0 | 0 | 0 | 0 | 0 | 0 | 0 | 0 | 0 | 0 | 0 | 0 | 0 | 0 | 0 | 0 | 0 | 0 | 0 | 0 |

**Table S8: Spring Tplank1**

| **POM** | **Aclau** | **Chelg** | **Chama** | **Eacut** | **PaPse** | **Pbrev** | **Tlong** | **Cuma** | **Mysi** | **Brach** | **Ncirr** | **Cyprr** | **Ammo** | **Chare** | **Calli** | **Gobbi** | **Llima** | **Mmerl** | **Sspra** | **Tlusc** | **Spilc** | **Pgunn** |
| --- | --- | --- | --- | --- | --- | --- | --- | --- | --- | --- | --- | --- | --- | --- | --- | --- | --- | --- | --- | --- | --- | --- |
| 0 | 1 | 1 | 1 | 1 | 1 | 1 | 1 | 1 | 1 | 1 | 1 | 0 | 1 | 1 | 0 | 0 | 1 | 1 | 1 | 1 | 0 | 1 |
| 0 | 0 | 0 | 0 | 0 | 0 | 0 | 0 | 0 | 1 | 1 | 0 | 0 | 0 | 1 | 1 | 1 | 1 | 1 | 1 | 1 | 1 | 1 |
| 0 | 0 | 0 | 0 | 0 | 0 | 0 | 0 | 0 | 1 | 1 | 0 | 0 | 0 | 1 | 1 | 1 | 1 | 1 | 1 | 1 | 1 | 1 |
| 0 | 0 | 0 | 0 | 0 | 0 | 0 | 0 | 0 | 1 | 1 | 0 | 0 | 0 | 1 | 1 | 1 | 1 | 1 | 1 | 1 | 1 | 1 |
| 0 | 1 | 0 | 0 | 0 | 0 | 0 | 0 | 0 | 1 | 1 | 0 | 0 | 0 | 1 | 1 | 1 | 1 | 1 | 1 | 1 | 1 | 1 |
| 0 | 0 | 0 | 0 | 0 | 0 | 0 | 0 | 0 | 1 | 1 | 0 | 0 | 1 | 1 | 1 | 1 | 1 | 1 | 1 | 1 | 1 | 1 |
| 0 | 0 | 0 | 0 | 0 | 0 | 0 | 0 | 0 | 1 | 1 | 0 | 0 | 0 | 1 | 1 | 1 | 1 | 1 | 1 | 0 | 1 | 1 |
| 0 | 1 | 0 | 0 | 0 | 0 | 0 | 0 | 0 | 1 | 1 | 0 | 0 | 0 | 1 | 1 | 1 | 1 | 1 | 1 | 0 | 1 | 1 |
| 0 | 0 | 0 | 0 | 0 | 0 | 0 | 0 | 0 | 0 | 1 | 0 | 0 | 0 | 1 | 0 | 1 | 0 | 1 | 0 | 0 | 1 | 1 |
| 0 | 0 | 0 | 0 | 0 | 0 | 0 | 0 | 0 | 0 | 1 | 0 | 0 | 0 | 1 | 0 | 1 | 0 | 1 | 0 | 1 | 1 | 1 |
| 0 | 0 | 0 | 0 | 0 | 0 | 0 | 0 | 0 | 0 | 1 | 0 | 0 | 0 | 1 | 0 | 1 | 0 | 1 | 0 | 0 | 1 | 1 |
| 0 | 1 | 1 | 1 | 0 | 1 | 1 | 1 | 1 | 1 | 1 | 0 | 0 | 1 | 1 | 0 | 1 | 1 | 1 | 1 | 0 | 0 | 1 |
| 0 | 0 | 0 | 0 | 0 | 0 | 0 | 0 | 0 | 1 | 0 | 0 | 0 | 1 | 1 | 0 | 1 | 0 | 1 | 1 | 0 | 0 | 1 |
| 0 | 0 | 0 | 0 | 0 | 0 | 0 | 0 | 0 | 0 | 0 | 0 | 0 | 0 | 0 | 0 | 0 | 0 | 0 | 0 | 0 | 0 | 0 |
| 0 | 0 | 0 | 0 | 0 | 0 | 0 | 0 | 0 | 0 | 0 | 0 | 0 | 0 | 0 | 0 | 0 | 0 | 0 | 0 | 0 | 0 | 0 |
| 0 | 0 | 0 | 0 | 0 | 0 | 0 | 0 | 0 | 0 | 0 | 0 | 0 | 0 | 0 | 0 | 0 | 0 | 0 | 0 | 0 | 0 | 0 |
| 0 | 0 | 0 | 0 | 0 | 0 | 0 | 0 | 0 | 0 | 0 | 0 | 0 | 0 | 0 | 0 | 0 | 0 | 0 | 0 | 0 | 0 | 0 |
| 0 | 0 | 0 | 0 | 0 | 0 | 0 | 0 | 0 | 0 | 0 | 0 | 0 | 0 | 0 | 0 | 0 | 0 | 0 | 0 | 0 | 0 | 0 |
| 0 | 0 | 0 | 0 | 0 | 0 | 0 | 0 | 0 | 0 | 0 | 0 | 0 | 0 | 0 | 0 | 0 | 0 | 0 | 0 | 0 | 0 | 0 |
| 0 | 0 | 0 | 0 | 0 | 0 | 0 | 0 | 0 | 0 | 0 | 0 | 0 | 0 | 0 | 0 | 0 | 0 | 0 | 0 | 0 | 0 | 0 |
| 0 | 0 | 0 | 0 | 0 | 0 | 0 | 0 | 0 | 0 | 0 | 0 | 0 | 0 | 0 | 0 | 0 | 0 | 0 | 0 | 0 | 0 | 0 |
| 0 | 0 | 0 | 0 | 0 | 0 | 0 | 0 | 0 | 0 | 0 | 0 | 0 | 0 | 0 | 0 | 0 | 0 | 0 | 0 | 0 | 0 | 0 |
| 0 | 0 | 0 | 0 | 0 | 0 | 0 | 0 | 0 | 0 | 0 | 0 | 0 | 0 | 0 | 0 | 0 | 0 | 0 | 0 | 0 | 0 | 0 |

**Table S9: Autumn TPlank0**

| **POM** | **Aclau** | **Chelg** | **Chama** | **Dangl** | **Eacut** | **Lwoll** | **PaPse** | **Tlong** | **Plong** | **Cuma** | **Gamm** | **Mysi** | **Brach** | **Chaet** | **Ammo** | **Gobbi** | **Syngn** | **Pmicro** | **Spilc** |
| --- | --- | --- | --- | --- | --- | --- | --- | --- | --- | --- | --- | --- | --- | --- | --- | --- | --- | --- | --- |
| 0 | 1 | 1 | 1 | 1 | 1 | 1 | 1 | 1 | 1 | 1 | 1 | 1 | 1 | 1 | 1 | 0 | 0 | 1 | 0 |
| 0 | 0 | 0 | 0 | 0 | 0 | 0 | 0 | 0 | 0 | 0 | 0 | 0 | 0 | 1 | 0 | 1 | 1 | 1 | 1 |
| 0 | 0 | 0 | 0 | 0 | 0 | 0 | 0 | 0 | 0 | 0 | 0 | 0 | 0 | 1 | 0 | 1 | 1 | 1 | 0 |
| 0 | 0 | 0 | 0 | 0 | 0 | 0 | 0 | 0 | 0 | 0 | 0 | 0 | 0 | 1 | 0 | 1 | 0 | 1 | 1 |
| 0 | 0 | 0 | 0 | 0 | 0 | 0 | 0 | 0 | 0 | 0 | 0 | 0 | 0 | 0 | 0 | 1 | 0 | 1 | 0 |
| 0 | 0 | 0 | 0 | 0 | 0 | 0 | 0 | 0 | 0 | 0 | 0 | 0 | 0 | 0 | 0 | 1 | 0 | 1 | 0 |
| 0 | 0 | 0 | 0 | 0 | 0 | 0 | 0 | 0 | 0 | 0 | 0 | 0 | 0 | 0 | 0 | 1 | 0 | 1 | 0 |
| 0 | 0 | 0 | 0 | 0 | 0 | 0 | 0 | 0 | 0 | 0 | 0 | 0 | 0 | 1 | 1 | 1 | 0 | 1 | 1 |
| 0 | 0 | 0 | 0 | 0 | 0 | 0 | 0 | 0 | 0 | 0 | 0 | 1 | 0 | 1 | 0 | 1 | 0 | 1 | 1 |
| 0 | 0 | 0 | 0 | 0 | 0 | 0 | 0 | 0 | 0 | 0 | 0 | 0 | 0 | 0 | 0 | 1 | 0 | 0 | 0 |
| 0 | 0 | 0 | 0 | 0 | 0 | 0 | 0 | 0 | 0 | 0 | 0 | 0 | 0 | 0 | 0 | 1 | 0 | 0 | 0 |
| 0 | 0 | 0 | 0 | 0 | 0 | 0 | 0 | 0 | 0 | 0 | 0 | 0 | 0 | 0 | 0 | 1 | 0 | 1 | 0 |
| 0 | 0 | 0 | 0 | 0 | 0 | 0 | 0 | 0 | 0 | 0 | 0 | 0 | 0 | 0 | 0 | 1 | 0 | 1 | 0 |
| 0 | 0 | 0 | 0 | 0 | 0 | 0 | 0 | 0 | 0 | 0 | 0 | 0 | 0 | 0 | 0 | 1 | 0 | 1 | 0 |
| 0 | 0 | 0 | 0 | 0 | 0 | 0 | 0 | 0 | 0 | 0 | 0 | 0 | 0 | 0 | 0 | 0 | 0 | 0 | 0 |
| 0 | 0 | 0 | 0 | 0 | 0 | 0 | 0 | 0 | 0 | 0 | 0 | 0 | 0 | 0 | 0 | 0 | 0 | 0 | 0 |
| 0 | 0 | 0 | 0 | 0 | 0 | 0 | 0 | 0 | 0 | 0 | 0 | 0 | 0 | 0 | 0 | 0 | 0 | 0 | 0 |
| 0 | 0 | 0 | 0 | 0 | 0 | 0 | 0 | 0 | 0 | 0 | 0 | 0 | 0 | 0 | 0 | 0 | 0 | 0 | 0 |
| 0 | 0 | 0 | 0 | 0 | 0 | 0 | 0 | 0 | 0 | 0 | 0 | 0 | 0 | 0 | 0 | 0 | 0 | 0 | 0 |
| 0 | 0 | 0 | 0 | 0 | 0 | 0 | 0 | 0 | 0 | 0 | 0 | 0 | 0 | 0 | 0 | 0 | 0 | 0 | 0 |

**Table S10: Autumn TPlank1**

| **POM** | **Aclau** | **Chelg** | **Chama** | **Dangl** | **Eacut** | **Lwoll** | **PaPse** | **Tlong** | **Plong** | **Cuma** | **Gamm** | **Mysi** | **Brach** | **Chaet** | **Ammo** | **Gobbi** | **Syngn** | **Pmicro** | **Spilc** |
| --- | --- | --- | --- | --- | --- | --- | --- | --- | --- | --- | --- | --- | --- | --- | --- | --- | --- | --- | --- |
| 0 | 1 | 1 | 1 | 1 | 1 | 1 | 1 | 1 | 1 | 1 | 1 | 1 | 1 | 1 | 1 | 0 | 1 | 1 | 0 |
| 0 | 0 | 0 | 0 | 1 | 0 | 1 | 0 | 0 | 0 | 0 | 0 | 1 | 1 | 1 | 0 | 1 | 1 | 1 | 1 |
| 0 | 0 | 0 | 0 | 0 | 0 | 0 | 0 | 0 | 0 | 0 | 0 | 1 | 1 | 1 | 0 | 1 | 1 | 1 | 1 |
| 0 | 0 | 0 | 0 | 1 | 0 | 0 | 0 | 0 | 0 | 0 | 0 | 1 | 1 | 1 | 0 | 1 | 1 | 1 | 1 |
| 0 | 0 | 0 | 0 | 0 | 0 | 0 | 0 | 0 | 0 | 0 | 0 | 1 | 1 | 1 | 0 | 1 | 1 | 1 | 1 |
| 0 | 1 | 0 | 0 | 1 | 0 | 1 | 0 | 0 | 0 | 0 | 0 | 1 | 1 | 1 | 0 | 1 | 1 | 1 | 1 |
| 0 | 0 | 0 | 0 | 0 | 0 | 0 | 0 | 0 | 0 | 0 | 0 | 0 | 1 | 1 | 0 | 1 | 1 | 1 | 1 |
| 0 | 0 | 0 | 0 | 1 | 0 | 1 | 0 | 0 | 0 | 0 | 0 | 1 | 1 | 1 | 1 | 1 | 1 | 1 | 1 |
| 0 | 1 | 0 | 0 | 0 | 0 | 0 | 0 | 0 | 0 | 0 | 0 | 1 | 1 | 1 | 0 | 1 | 1 | 1 | 1 |
| 0 | 0 | 0 | 0 | 0 | 0 | 0 | 0 | 0 | 0 | 0 | 0 | 0 | 1 | 1 | 0 | 1 | 0 | 1 | 1 |
| 0 | 0 | 0 | 0 | 0 | 0 | 0 | 0 | 0 | 0 | 0 | 0 | 0 | 1 | 1 | 0 | 1 | 0 | 1 | 1 |
| 0 | 0 | 0 | 0 | 1 | 0 | 0 | 0 | 0 | 0 | 0 | 0 | 0 | 1 | 1 | 0 | 1 | 0 | 1 | 1 |
| 0 | 0 | 0 | 0 | 0 | 0 | 0 | 0 | 0 | 0 | 0 | 0 | 0 | 1 | 1 | 0 | 1 | 1 | 1 | 1 |
| 0 | 0 | 0 | 0 | 0 | 0 | 0 | 0 | 0 | 0 | 0 | 0 | 0 | 1 | 1 | 0 | 1 | 0 | 1 | 1 |
| 0 | 0 | 0 | 0 | 0 | 0 | 0 | 0 | 0 | 0 | 0 | 0 | 0 | 0 | 0 | 0 | 0 | 0 | 0 | 0 |
| 0 | 0 | 0 | 0 | 0 | 0 | 0 | 0 | 0 | 0 | 0 | 0 | 0 | 0 | 0 | 0 | 0 | 0 | 0 | 0 |
| 0 | 0 | 0 | 0 | 0 | 0 | 0 | 0 | 0 | 0 | 0 | 0 | 0 | 0 | 0 | 0 | 0 | 0 | 0 | 0 |
| 0 | 0 | 0 | 0 | 0 | 0 | 0 | 0 | 0 | 0 | 0 | 0 | 0 | 0 | 0 | 0 | 0 | 0 | 0 | 0 |
| 0 | 0 | 0 | 0 | 0 | 0 | 0 | 0 | 0 | 0 | 0 | 0 | 0 | 0 | 0 | 0 | 0 | 0 | 0 | 0 |
| 0 | 0 | 0 | 0 | 0 | 0 | 0 | 0 | 0 | 0 | 0 | 0 | 0 | 0 | 0 | 0 | 0 | 0 | 0 | 0 |

# **Supplementary Section S4**

# **« Stable isotopes mixing models (IsoWeb) »**

We used a combination of two mixing models to identify drivers of variation in energy pathways for main consumers as proposed by Giraldo et al., (2017). The first model, IsoWeb (Kadoya et al., 2012) is a food-web-scale model based on SI data with a topological description of the food-web given *a priori (previously reported as TPlank1*on the Supplementary Tables S5 to S10). One of the main advantages of IsoWeb is that it allows for TEF variation across links, assuming that TEFs follow a normal distribution with a mean of 0.8 for carbon and 2.3 for nitrogen, as proposed for zooplankton food-webs (de Figueiredo et al., 2020; Schwamborn and Giarrizzo, 2015). As a result, the model provides predator-prey TEF estimates for both C and N (Supplementary Fig. S14). Models were run seasonally with the following parameters: 10^6^ chain length, 50k burn-ins, and thin number 500 for three parallel Markov Chain Monte Carlo (MCMC) chains. Convergence was assessed using the Gelman-Rubin test (Gelman et al., 2014).


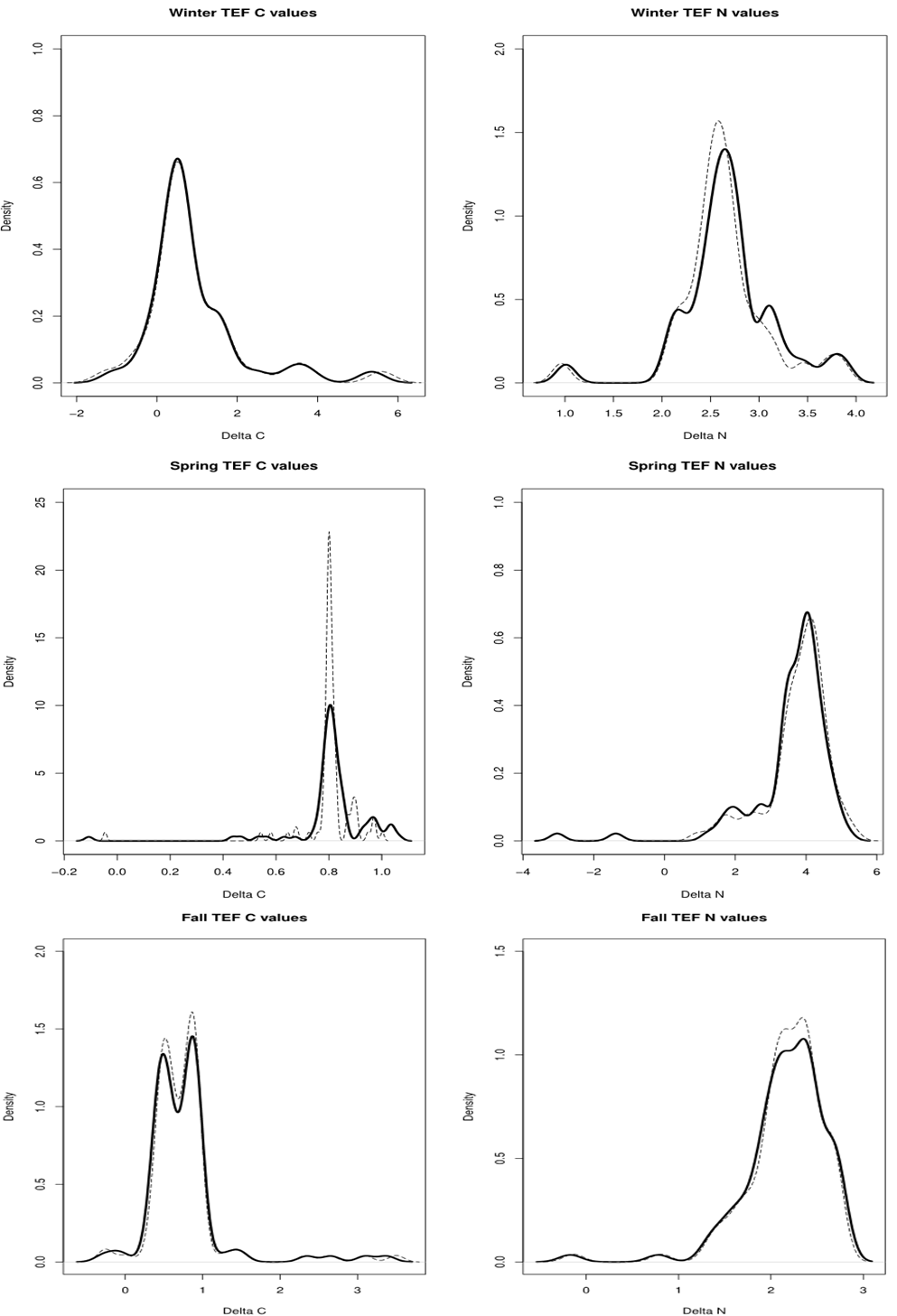


**Figure S14: Posterior probability density plots of seasonal (winter, spring and autumn) trophic enrichment factors (TEF) for carbon (left) and nitrogen (right) as estimated by the IsoWeb model.** Black lines illustrate the mean value after 10^6 iterations, and dotted lines the median value.

# **Supplementary Section S5**

# **« MixSIAR Results »**

**Table S11: Summary results of MixSIAR models for predatory plankton.** The posterior distribution includes mean values, standard deviation and 25%, 50%, 75% and 97.5% credible intervals (CI).

|  | **Mean** | **SD** | **25%CI** | **50%CI** | **75%CI** | **97.5%CI** |
| --- | --- | --- | --- | --- | --- | --- |
| **Autumn** |  |  |  |  |  |  |
| **Chaetognaths** |  |  |  |  |  |  |
| POM | 0.07 | 0.05 | 0.03 | 0.06 | 0.10 | 0.20 |
| < 1mm | 0.06 | 0.05 | 0.02 | 0.04 | 0.08 | 0.19 |
| 1 - 1.5 mm | 0.46 | 0.12 | 0.38 | 0.47 | 0.54 | 0.66 |
| 1.5 - 2mm | 0.14 | 0.09 | 0.08 | 0.13 | 0.19 | 0.35 |
| > 2mm | 0.28 | 0.11 | 0.20 | 0.26 | 0.34 | 0.53 |
| **Sardine** |  |  |  |  |  |  |
| < 1mm | 0.13 | 0.09 | 0.06 | 0.12 | 0.19 | 0.34 |
| 1 - 1.5 mm | 0.49 | 0.14 | 0.40 | 0.51 | 0.59 | 0.72 |
| 1.5 - 2mm | 0.18 | 0.12 | 0.09 | 0.15 | 0.24 | 0.47 |
| > 2mm | 0.20 | 0.10 | 0.13 | 0.18 | 0.25 | 0.47 |
| **Spring** |  |  |  |  |  |  |
| **Callionymus spp** |  |  |  |  |  |  |
| < 1mm | 0.03 | 0.03 | 0.01 | 0.02 | 0.04 | 0.11 |
| 1 - 1.5 mm | 0.37 | 0.13 | 0.29 | 0.38 | 0.47 | 0.64 |
| 1.5 - 2mm | 0.37 | 0.14 | 0.27 | 0.36 | 0.47 | 0.67 |
| > 2mm | 0.22 | 0.13 | 0.12 | 0.21 | 0.31 | 0.53 |
| **Herring** |  |  |  |  |  |  |
| POM | 0.04 | 0.04 | 0.01 | 0.03 | 0.06 | 0.15 |
| < 1mm | 0.08 | 0.05 | 0.05 | 0.07 | 0.11 | 0.20 |
| 1 - 1.5 mm | 0.18 | 0.09 | 0.11 | 0.17 | 0.23 | 0.37 |
| 1.5 - 2mm | 0.27 | 0.13 | 0.17 | 0.25 | 0.35 | 0.56 |
| > 2mm | 0.44 | 0.14 | 0.35 | 0.44 | 0.53 | 0.69 |
| **Dab** |  |  |  |  |  |  |
| POM | 0.02 | 0.02 | 0.01 | 0.02 | 0.03 | 0.08 |
| < 1mm | 0.03 | 0.02 | 0.01 | 0.02 | 0.04 | 0.07 |
| 1 - 1.5 mm | 0.18 | 0.10 | 0.11 | 0.17 | 0.25 | 0.40 |
| 1.5 - 2mm | 0.11 | 0.06 | 0.07 | 0.11 | 0.15 | 0.24 |
| > 2mm | 0.66 | 0.09 | 0.59 | 0.66 | 0.72 | 0.82 |
| **Mysida** |  |  |  |  |  |  |
| POM | 0.07 | 0.06 | 0.02 | 0.05 | 0.09 | 0.22 |
| < 1mm | 0.15 | 0.09 | 0.08 | 0.14 | 0.20 | 0.35 |
| 1 - 1.5 mm | 0.12 | 0.07 | 0.07 | 0.12 | 0.16 | 0.27 |
| 1.5 - 2mm | 0.37 | 0.16 | 0.26 | 0.35 | 0.47 | 0.70 |
| > 2mm | 0.29 | 0.16 | 0.17 | 0.30 | 0.41 | 0.55 |
| **Sprat** |  |  |  |  |  |  |
| < 1mm | 0.24 | 0.11 | 0.16 | 0.23 | 0.31 | 0.48 |
| 1 - 1.5 mm | 0.27 | 0.12 | 0.17 | 0.26 | 0.35 | 0.53 |
| 1.5 - 2mm | 0.36 | 0.13 | 0.26 | 0.36 | 0.45 | 0.64 |
| > 2mm | 0.13 | 0.10 | 0.05 | 0.11 | 0.19 | 0.37 |
| **Winter** |  |  |  |  |  |  |
| **Chae-gnathe** |  |  |  |  |  |  |
| POM | 0.09 | 0.06 | 0.04 | 0.08 | 0.12 | 0.24 |
| 1 - 1.5 mm | **0.51** | **0.13** | **0.42** | **0.52** | **0.60** | **0.74** |
| 1.5 - 2mm | 0.17 | 0.11 | 0.08 | 0.16 | 0.24 | 0.41 |
| > 2mm | 0.24 | 0.12 | 0.14 | 0.22 | 0.31 | 0.50 |
| **Herring** |  |  |  |  |  |  |
| POM | 0.05 | 0.04 | 0.02 | 0.04 | 0.08 | 0.14 |
| 1 - 1.5 mm | **0.60** | **0.09** | **0.54** | **0.61** | **0.66** | **0.77** |
| 1.5 - 2mm | 0.15 | 0.10 | 0.05 | 0.14 | 0.23 | 0.37 |
| > 2mm | 0.20 | 0.11 | 0.12 | 0.19 | 0.28 | 0.42 |
| **Mysida** |  |  |  |  |  |  |
| POM | 0.13 | 0.09 | 0.07 | 0.12 | 0.19 | 0.33 |
| 1 - 1.5 mm | **0.33** | **0.14** | **0.23** | **0.33** | **0.43** | **0.62** |
| 1.5 - 2mm | 0.32 | 0.16 | 0.20 | 0.32 | 0.43 | 0.64 |
| > 2mm | 0.21 | 0.15 | 0.09 | 0.19 | 0.31 | 0.54 |
| **Plaice** |  |  |  |  |  |  |
| POM | 0.07 | 0.05 | 0.03 | 0.06 | 0.10 | 0.19 |
| 1 - 1.5 mm | **0.82** | **0.08** | **0.77** | **0.83** | **0.87** | **0.95** |
| 1.5 - 2mm | 0.05 | 0.05 | 0.01 | 0.03 | 0.07 | 0.17 |
| > 2mm | 0.06 | 0.06 | 0.02 | 0.05 | 0.09 | 0.21 |
| **Sardine** |  |  |  |  |  |  |
| 1 - 1.5 mm | **0.51** | **0.15** | **0.41** | **0.52** | **0.62** | **0.79** |
| 1.5 - 2mm | 0.19 | 0.13 | 0.09 | 0.17 | 0.27 | 0.50 |
| > 2mm | 0.30 | 0.15 | 0.19 | 0.28 | 0.39 | 0.62 |

## Diet by species :

**Preliminary information** : Dealing with too many sources in MixSIAR poses challenges due to increased model complexity, potential overfitting, difficulty in discriminating between similar sources, and collinearity issues. These factors can lead to longer computation times, less reliable estimates of source contributions, and increased uncertainty in the results. In this study, a large number of predator-prey interactions (up to 13 for Sardine in Autumn) were found for some species in Spring and Autumn. This resulted in certain species sharing similar stable isotopes, leading to an undetermined system. Therefore, caution should be exercised when interpreting the specific contributions of these species to the mixing model. Despite this limitation, the results obtained by pooling species based on size-classes (the main focus of this study) rather than a taxonomic approach remain valid and provide valuable insights into the contributions from different functional groups to planktonic food-webs

**Winter :** In herring, diet was largely dominated by *A. clausi*, representing 58% of the diet, followed by Paracalanus/Pseudocalanus (24%). However, the correlation value among these prey was extremely high (-0.89) suggesting that either one of these sources could potentially dominate the diet. Among other carnivorous plankton, mysids and chaetognaths were also frequently encountered. Mysids appeared as omnivorous, feeding mainly on *T. longicornis* (32%), with all other prey (including POM) representing between 14 to 22% of the diet. For chaetognaths, the main prey was *A. clausi* (30%), and POM contributed less than 10%, suggesting that chaetognaths are mostly carnivores.

**Spring:** Herring larvae (16 ± 3.8 mm SL) consumed a wide variety of species, but with a clear dominance of *C. helgolandicus* that accounted for 44% to their diet. To a lesser extent, *T. longicornis* (13%) and *A. clausi* (11%) were also minor contributors. A similar pattern was found for dab larvae (10 ± 2.6 mm SL), where *C. helgolandicus* also dominated the diet at 66%, followed by *A. clausi* (15%). All other species contributed by less than 10%. Sprat larvae (20 ± 3.5% mm SL) consumed a wide variety of species with no clear dominance of any single prey (each species accounting for ~10%). Similarly, Callionymus larvae also had a generalist diet with no clear dominance of any single prey (*A. clausi* and *C. helgolandicus* accounting for ~23% each). Mysids were also frequently encountered in spring, and displayed a similar feeding pattern to herring and dab, feeding mainly on *C. helgolandicus* (29%), *T. longicornis* (18%) and *C. hamatus* (13%), with a minor contribution from *A. clausi* (5%).

**Autumn:** Sardine spawning in the EEC extends from May to December so larvae of similar sizes (when compared to winter individuals) were found during the autumn (~21 mm SL). The large number of original sources (n = 13) resulted in a number of species sharing similar stable isotope and size values (Permanova followed by pair-wise comparisons). These were C*. helgolandicus* and *T. longicornis* (“Chel_Plo”, p = 0.77), *D. anglicus* and *Para-Pseudocalanus* (“Dan_PaPs”, p = 0.85), as well as zoe brachura with gamaridae (p = 0.28) and *L. wollastoni* (p= 0.19 and 0.72 respectively). Because the latter species might have different functional roles in the plankton, only zoe brachyura and gammaridae were combined under “Malacostraca” and the copepod *L. wollastoni* was kept separate. Results of the MixSIAR model indicate that main contributors to sardine diet were *D. angelicus* and *Para-Pseudocalanus* representing 34% of the diet, followed by *A. Clausi* (15%), *E. acutifrons* (13%) and *C. hamatus* (13%). All other prey groups were negligible and contributed to less than 10%. Chaetognaths (~7-8 mm TL) were frequently encountered in autumn and winter. Some prey *C. helgolandicus* and *T. longicornis* (“Chel_Tlo”, p = 0.78), *D. anglicus* and *Para-Pseudocalanus* (“Dan_PaPs”, p = 0.86), as well as zoe brachura with gammaridae (“Malacostraca” p = 0.25) had similar isotopic signatures and were combined *a priori*. They consumed a wide variety of prey in similar proportions (slight dominance of *A. clausi* at 34%).
